# Distinct transcriptomic changes in E14.5 mouse skeletal muscle lacking RYR1 or Ca_v_1.1 converge at E18.5

**DOI:** 10.1371/journal.pone.0194428

**Published:** 2018-03-15

**Authors:** Dilyana Filipova, Margit Henry, Tamara Rotshteyn, Anna Brunn, Mariana Carstov, Martina Deckert, Jürgen Hescheler, Agapios Sachinidis, Gabriele Pfitzer, Symeon Papadopoulos

**Affiliations:** 1 Institute of Vegetative Physiology, Center of Physiology and Pathophysiology, University of Cologne, Cologne, Germany; 2 Institute of Neurophysiology and Center for Molecular Medicine Cologne (CMMC), University of Cologne, Cologne, Germany; 3 Department of Neuropathology, University of Cologne, Cologne, Germany; Cinvestav-IPN, MEXICO

## Abstract

In skeletal muscle the coordinated actions of two mechanically coupled Ca^2+^ channels—the 1,4-dihydropyridine receptor (Ca_v_1.1) and the type 1 ryanodine receptor (RYR1)–underlie the molecular mechanism of rapid cytosolic [Ca^2+^] increase leading to contraction. While both [Ca^2+^]_i_ and contractile activity have been implicated in the regulation of myogenesis, less is known about potential specific roles of Ca_v_1.1 and RYR1 in skeletal muscle development. In this study, we analyzed the histology and the transcriptomic changes occurring at E14.5 –the end of primary myogenesis and around the onset of intrauterine limb movement, and at E18.5 –the end of secondary myogenesis, in WT, RYR1^-/-^, and Ca_v_1.1^-/-^ murine limb skeletal muscle. At E14.5 the muscle histology of both mutants exhibited initial alterations, which became much more severe at E18.5. Immunohistological analysis also revealed higher levels of activated caspase-3 in the Ca_v_1.1^-/-^ muscles at E14.5, indicating an increase in apoptosis. With WT littermates as controls, microarray analyses identified 61 and 97 differentially regulated genes (DEGs) at E14.5, and 493 and 1047 DEGs at E18.5, in RYR1^-/-^ and Ca_v_1.1^-/-^ samples, respectively. Gene enrichment analysis detected no overlap in the affected biological processes and pathways in the two mutants at E14.5, whereas at E18.5 there was a significant overlap of DEGs in both mutants, affecting predominantly processes linked to muscle contraction. Moreover, the E18.5 vs. E14.5 comparison revealed multiple genotype-specific DEGs involved in contraction, cell cycle and miRNA-mediated signaling in WT, neuronal and bone development in RYR1^-/-^, and lipid metabolism in Ca_v_1.1^-/-^ samples. Taken together, our study reveals discrete changes in the global transcriptome occurring in limb skeletal muscle from E14.5 to E18.5 in WT, RYR1^-/-^ and Ca_v_1.1^-/-^ mice. Our results suggest distinct functional roles for RYR1 and Ca_v_1.1 in skeletal primary and secondary myogenesis.

## Introduction

Skeletal muscle is the largest organ in the vertebrate body. Although the most prominent association coming into mind when one thinks of skeletal muscle is probably that related to contraction and movement, the functional repertoire of this organ is by far more versatile. For instance, involvement in energy metabolism, myokine secretion and further mechanisms of crosstalk with various organs are additional important roles of skeletal muscle [1,2]. Muscle tissue itself shows a high degree of heterogeneity and additional cell types like neurons, smooth muscle and blood cells, as well as fibroblasts and connective tissue contribute to the overall structural and functional characteristics of a certain muscle [3]. Accordingly, the proper formation of the skeletal muscle organ is a complex, only partially understood multistep process, subjected to a strict spatiotemporal regulation throughout development [4].

In mice the formation of skeletal muscle begins around embryonic day E8.5 / E9, when somites differentiate into the sclerotome and dermomyotome, and proceeds until birth (E18.5 –E19) with a subsequent postnatal maturation for 2–3 weeks [5]. Prenatally, skeletal muscle development can be divided into embryonic (from E8.5 until E14.5) and fetal (E14.5 to E18.5 / birth). During the embryonic phase initial myogenic events give rise to the primary myotome and myogenic precursor cells, the latter differentiating into embryonic myoblasts that invade the myotome and fuse into myotubes. Simultaneously, myogenic progenitors migrate from the dermomyotome to the limbs and differentiate into primary multinucleated fibers in a process described as primary myogenesis [6]. In the fetal phase, secondary myogenesis takes place whereby fetal myoblasts, derived from Pax3/Pax7 positive muscle progenitor cells, merge with each other or with primary fibers to form secondary muscle fibers [7,8]. Thus, the primary myogenesis lays the foundation of the developing skeletal muscle and during the secondary myogenesis the muscle grows and differentiates. In line with this notion, a genome wide expression analysis has shown that embryonic and fetal myoblasts have distinct transcriptomic profiles [9]. The proper transition between the different stages in myogenesis is regulated by the strict spatiotemporal expression of canonical myogenic regulatory factors (MRFs) [10] and involves a crosstalk with the surrounding connective and neuronal tissue [4]. Around the time of late embryonic and early fetal development the first skeletal muscle contractions effective in limb movement start appearing in mouse [11].

In skeletal muscle contractions are initiated by action potentials originating from the motor neurons that induce a depolarization wave travelling along deep invaginations of the sarcolemma—the T tubuli. The signal is then transmitted to the sarcoplasmic reticulum (SR)–the major Ca^2+^ reservoir in skeletal muscle, leading to a rapid local Ca^2+^ release from the SR and a high increase of the cytosolic Ca^2+^ concentration [Ca^2+^]_i_, which enables muscle contraction—a process known as excitation-contraction coupling (ECC) [12,13]. The released Ca^2+^ participates in a wide variety of signaling pathways and during development is involved in myoblast migration and fusion, as well as in muscle terminal differentiation and growth [14,15]. Additionally muscle contraction, by means of mechanotransduction, triggers downstream cascades which temporary overlap with Ca^2+^-induced signaling responses and which play a crucial role in skeletal muscle’s development and adaptation to exercise [16].

On a molecular level ECC in skeletal muscle can be described as conformational signal transmission between two functionally and, most likely, also mechanically coupled Ca^2+^ channels, the sarcolemmal / t-tubular, voltage-gated Ca_v_1.1 (also known as 1,4-dihydropyridine receptor, DHPR)and the Ca^2+^ release channel ryanodine receptor type 1 (RYR1), anchored in the SR membrane. Ca_v_1.1 acts as a voltage sensor who upon membrane depolarization imposes conformational changes on RYR1, causing the latter to release Ca^2+^ from the SR into the sarcoplasm [17]. In the context of ECC the structural, electrophysiological and biochemical characteristics of both Ca_v_1.1 and RYR1 have been the subject of numerous investigations [17–21]. Mutations in these channels have been linked to the pharmacogenetic condition of malignant hyperthermia, to hypokalemic periodic paralysis, and to a spectrum of myopathies [22]. The often prenatal onset of the latter [23] implies important functions of the two Ca^2+^ channels in myogenesis.

Two mouse models have been utilized for studying the functions of RYR1 and DHPR—the *dyspedic* mouse, a RYR1 null mutant model (RYR1^-/-^) [24]; and the *dysgenic* mouse—a null mutant for the principal voltage-sensor-containing subunit of the DHPR—Ca_v_1.1 (Ca_v_1.1^-/-^) [25]. While both heterozygous RYR1^+/-^ and Ca_v_1.1^+/-^ mutants are allegedly indistinguishable from their WT littermates, the homozygous RYR1^-/-^ and Ca_v_1.1^-/-^ mutants cannot support ECC and die at birth from asphyxia [24,26,27]. We have previously shown that at E18.5 the RYR1^-/-^ fetuses exhibit extensive changes in limb skeletal muscle gene expression, affecting major signaling pathways like the mitogen-activated protein kinase (MAPK), Wnt and PI3K-AKT pathways, as well as multiple genes related to muscle structure and function [28]. The wide spectrum of gene expression alterations associated with the complete absence of RYR1 in deed suggested a critical role of this channel in myogenesis. However, the relatively late stage (E18.5) at which we performed the expression analysis probably represented not only expression changes directly related to RYR1 absence, but most likely also comprised a reactive and/or degenerative component.

Here, we examined how the absence of RYR1 or Ca_v_1.1 affects primary and secondary myogenesis at E14.5 and E18.5, respectively. For both stages we compare the body gross morphology as well the structural features of WT (RYR1^+/+^ and Ca_v_1.1^+/+^), heterozygous (RYR1^+/-^ and Ca_v_1.1^+/-^) and homozygous (RYR1^-/-^ and Ca_v_1.1^-/-^) limb skeletal muscle from littermates. Muscle development was mildly delayed in RYR1^-/-^, Ca_v_1.1^+/-^ and Ca_v_1.1^-/-^ fetuses at E14.5 as indicated by the higher degree of fascicular disorganization in these animals. Much more severe changes were observed at E18.5, at which stage the limb skeletal muscle of RYR1^-/-^ and Ca_v_1.1^-/-^ fetuses consisted almost exclusively of undifferentiated myotubes and amorphous tissue. Structural disorganization was also observed in Ca_v_1.1^+/-^ skeletal muscle at E18.5. Expression analysis of MRFs revealed lower mRNA levels for *Six1* in RYR1^-/-^ and for *Mrf4* in Ca_v_1.1^-/-^ at E14.5 and of *Pax3* in RYR1^-/-^ at E18.5. At E14.5 RYR1^-/-^ skeletal muscle exhibited high expression levels of an embryonic Ca_v_1.1 splice variant, Δ29, indicating that the presence of RYR1 impacts the advancement of the myogenic schedule.

We performed microarray analyses (MAs) using RNA isolated from limb skeletal muscle at E14.5 or E18.5 to characterize the transcriptome of RYR1^-/-^ and Ca_v_1.1^-/-^ mice versus their respective WT littermates. While we find almost no overlap in the transcriptomic changes (compared to WT) of RYR1^-/-^ and Ca_v_1.1^-/-^ samples at E14.5, at E18.5 we reveal a significant convergence of differential gene expression in both mutants, with common DEGs primarily associated with muscle contraction. However, our analysis also reveals processes and structures which are affected in a mutant-specific manner, e.g., the extracellular matrix (ECM) in RYR1^-/-^ and lipid metabolism in Ca_v_1.1^-/-^ skeletal muscle. Finally, we find further indications that both mutants recapitulate only the incomplete myogenic program as the amount of DEGs, microRNAs and regulated processes, when going from E14.5 to E18.5, is by far greater in samples from WT skeletal muscle.

## Materials and methods

### Ethics statement

Animal experiments were carried out in accordance with the guidelines of the European Commission (Directive 2010/63/EU) and of the German animal welfare act (TierSchG). The mice were housed in the Animal Facility of the Center for Molecular Medicine Cologne (CMMC), a part of the Medical Faculty of the University of Cologne according to the European Union Recommendation 2007/526/EG. All experimental protocols were approved by the local governmental authorities (Landesamt für Natur, Umwelt und Verbraucherschutz, North Rhine-Westphalia, 84–02.04.2015.A054). Effort was taken to minimize animal suffering.

### Animals and skeletal muscle preparation

Both the RYR1^+/-^ *dyspedic* (ry1_42_) and the Ca_v_1.1^+/-^ *dysgenic* mouse (*mdg*) lines, were from the C57BL/6J background [27,29]. Six heterozygous RYR1^+/-^ or Ca_v_1.1^+/-^ male and female mice were subjected to timed mating (pairing was only among lines: either RYR1^+/-^ x RYR1^+/-^ or Ca_v_1.1^+/-^ x Ca_v_1.1^+/-^). Three pregnant females of each line were sacrificed at day 14.5 and three at day 18.5 post coitum by cervical dislocation and each fetus was prepared and handled separately [28]. Skeletal muscle from the front and hind limbs of each fetus was dissected as previously described [28], pooled for each animal in RNAlater (Cat. No. 76104, Qiagen, Hilden, Germany) on ice during sample collection and centrifuged for 10 min at 16,000 x g. The RNAlater was then removed and the samples were immediately frozen in liquid nitrogen and stored at -80 °C until use.

The fetuses were genotyped via PCR as described below. From each litter the limb skeletal muscle samples from one WT, one heterozygous (either RYR1^+/-^ or Ca_v_1.1^+/-^) and one homozygous (either RYR1^-/-^ or Ca_v_1.1^-/-^) mutant littermate were used in the subsequent analyses (n = 3 biological replicates = 3 animals for each group).

### Genotyping

A small terminal segment from the tail of each fetus was lysed in 100 μl lysis buffer (25 mM NaOH, 0.1 mM EDTA) at 95 °C for 30 minutes, followed by an addition of 100 μl ice-cold neutralization buffer (40 mM Trizma-HCl) on ice. One μl of each sample was used as a template for genotyping PCRs using the DreamTaq Polymerase (Thermo Scientific, Cat. #EP0703) as per manufacturer’s instructions. For genotyping the RYR1 line *(Ryr1* gene), primers forward: 5’- GGACTGGCAAGAGGACCGGAGC -3’ and reverse: 5’-GGAAGCCAGGGCTGCAGGTGAGC-3’ were used for detection of the WT (+) allele; and primers forward: 5’-GGACTGGCAAGAGGACCGGAGC -3’ and reverse: 5’-CCTGAAGAACGAGATCAGCAGCCTCTGTCCC-3’–for the detection of the mutant (-) allele. Primers forward: 5’-GCTTTGCAGATGTTCGGGAAGATCGCCATGG-3’ and reverse: 5’-GCAGCTTTCCACTCAGGAGGGATCCAGTGT-3’ were used for genotyping the Ca_v_1.1 line (*Cacna1s* gene), the resulting PCR products being subsequently subjected to a restriction analyses via *Ear*I (NEB, Cat. #R0528S). *Ear*I digests only the PCR product from the WT *Ca*_*v*_*1*.*1* allele but not the mutant allele. PCR products and *Ear*I digestions were analyzed via runs on 2% agarose gels.

### Morphological analyses

Comparison of the overall morphology, body shape and size of littermates from different genotypes, was carried out after taking whole-body photographs of animal fetuses (n = 3) at E14.5 and E18.5 of each of the following genotypes: RYR1^+/+^ (WT), RYR1^+/-^, RYR1^-/-^; Ca_v_1.1^+/+^ (WT), Ca_v_1.1^+/-^, and Ca_v_1.1^-/-^. Representative photographs from each group are shown.

### Histology and immunohistochemistry

The entire hind limbs of E14.5 and E18.5 fetuses were prepared and mounted on thick filter paper with Tissue-Tek OCT compound (Miles Scientific, Naperville, IL), snap-frozen in isopentane (Fluka, Neu-Ulm, Germany) pre-cooled by dry ice, and stored at −80°C until preparation of serial 10 μm frozen sections. Sections were stained with H&E. Immunohistochemistry to detect apoptosis was performed with monoclonal rabbit anti-mouse activated caspase-3 (clone C92-605; BD Biosciences, Heidelberg, Germany) by use of the avidin-biotin complex technique with appropriate biotinylated secondary antibodies (Vectastain Elite Kit; Vector Laboratories, Burlingame, CA). Peroxidase reaction product was visualized using 3,3’-diaminobenzidine (Sigma-Aldrich) as chromogene and H_2_O_2_ as co-substrate.

### RNA extraction

Total skeletal muscle RNA was extracted from limb skeletal muscles as described previously [28]. Briefly, the muscle tissue was rapidly homogenized mechanically via a steel micropestle (Cat. #6–1062, neoLab, Heidelberg, Germany) in liquid nitrogen. Total RNA was extracted with the *Maxwell 16 LEV simplyRNA Tissue Kit* (Cat. #AS1280, Promega, Madison, WI) using a Maxwell 16 instrument (Cat. #AS2000, Promega, Madison, WI) according to the manufacturer’s instructions. RNA concentration was measured with a NanoDrop 1000 Spectrophotometer (Peqlab, Erlangen, Germany). 250 or 500 ng of each RNA sample were analyzed via runs on 2% agarose gels next to 2 μl of RiboRuler High Range RNA Ladder (Cat. # SM1821, ThermoFisher Scientific, Hagen, Germany).

### cDNA synthesis and quantitative real-time PCRs (qRT-PCRs)

1 μg total RNA of each sample was used for cDNA synthesis via the QuantiTect^®^ Reverse Transcription Kit (Cat. #205311 Qiagen, Hilden, Germany) according to the manufacturer’s instructions. Samples were eluted in a final volume of 500 μl nuclease-free water. qRT-PCR analyses were applied for determination of the relative gene expression levels of selected genes as previously described [28]. All primers (Table 1) were designed using the Primer-BLAST[30] online tool (NCBI, www.ncbi.nlm.nih.gov/tools/primer-blast/) with a Tm range of 58 °C–60 °C, an optimal length of 20 bases and an amplicon of 105–115 bp, and were purchased from Sigma Aldrich (Munich, Germany). The qRT-PCR reaction mixtures were prepared via the GoTaq^®^ qPCR Master Mix kit (Cat. #A6001, Promega, Madison, WI) and relative expression levels were calculated as fold change (FC) using the 2^−ΔΔCt^ method as previously described [28] with the *Cytb* transcript as endogenous control.

**Table 1:** Primers sequences and amplicon size used in qRT-PCR analyses.

Gene	Primers (5’ to 3’)	Amplicon (bp)
*Abra*	Fwd: GCCCCCAAAACTCTGTCTCC	111
	Rev: GACAACCGTTCTGGTCACCT	
*Actb*	Fwd: GCCTCACTGTCCACCTTCCA	115
	Rev: AAAACGCAGCTCAGTAACAGTC	
*Ankrd1*	Fwd: CCTGCGAGGCTGATCTCAAT	110
	Rev: CGCACCGAAGGTCATCAAGA	
*Cacna1s* exons 10–11	Fwd: GCCACTCTGGTTGACCCATT	115
	Rev: GGACATGAAGTACTGGCGCA	
*Cdh3*	Fwd: CAACGAAGCCCCTGTGTTTG	109
	Rev: CTCCTTGTCTGGGTCCTGTG	
*Col19a1*	Fwd: TTGGATTGCCAGGAGAACAT	114
	Rev: CAGCATCACCCTTCAGACCT	
*Creb5*	Fwd: AGGGAGTTGAAGGCTACTGGA	107
	Rev: TCTGCAGCTCCGACCTATCT	
*Cytb*	Fwd: CCATTCTACGCTCAATCCCCA	109
	Rev: AGGCTTCGTTGCTTTGAGGT	
*Derl3*	Fwd: ATGCTCTTCGTGTTCCGCTA	109
	Rev: GCAGAGTCATAAGAACACCACC	
*Eda2r*	Fwd: AGAGGATGGATTTGATCTGTTGTTG	106
	Rev: AAGGCAGTTGTCACGCTCTC	
*Fn1*	Fwd: GGTTCGGGAAGAGGTTGTGA	105
	Rev: ATGGCGTAATGGGAAACCGT	
*Fos*	Fwd: AGTCAAGGCCTGGTCTGTGT	100
	Rev: TCCAGCACCAGGTTAATTCC	
*Gapdh*	Fwd: AGTGTTTCCTCGTCCCGTAG	119
	Rev: TGATGGCAACAATCTCCACT	
*Hbb-y*	Fwd: TTGGCTAGTCACTTCGGCAAT	107
	Rev: AGGGCTCAGTGGTACTTGTG	
*Hdac4*	Fwd: CCAATGCCAATGCTGTCCAC	112
	Rev: TGCGCCTCAATCAGAGAGTG	
*Irx2*	Fwd: GTCTACACGTCGACTCGCTC	107
	Rev: ACACTCTGAGCCTGATTCGC	
*Klf4*	Fwd: TACCCCTACACTGAGTCCCG	110
	Rev: GGAAAGGAGGGTAGTTGGGC	
*Mcpt4*	Fwd: GTGGGCAGTCCCAGAAAGAA	107
	Rev: GCATCTCCGCGTCCATAAGA	
*Mlip*	Fwd: AAGCATGAACCAGGAAGCTCA	114
	Rev: CTGGACCCTCTCTTGTTTGCT	
*Mrf4*	Fwd: GCAGAGGGCTCTCCTTTGTA	105
	Rev: AACGTGTTCCTCTCCACTGC	
*Mybpc2*	Fwd: ACACTGAACATCCGCCGAC	113
	Rev: TGTGGCACTCGGACATCCA	
*Myf5*	Fwd: GAAGGTCAACCAAGCTTTCG	109
	Rev: GCTCTCAATGTAGCGGATGG	
*Myl2*	Fwd: AAAGAGGCTCCAGGTCCAAT	105
	Rev: CACCTTGAATGCGTTGAGAA	
*Mylpf*	Fwd: ATAACCCCAGAAGAACTGCTCC	108
	Rev: TTCTCTTGGCCTTCTTGGGTG	
*Myod*	Fwd: GGCTACGACACCGCCTACTA	110
	Rev: GTGGAGATGCGCTCCACTAT	
*Myog*	Fwd: CTGCACTCCCTTACGTCCAT	103
	Rev: CCCAGCCTGACAGACAATCT	
*Nefl*	Fwd: TTCAGGATCTATGGCAATGTGA	115
	Rev: TCCCATGAGGTTGCACATGAA	
*Nell1*	Fwd: ATCAGAGGAAGGCGTTTGGG	111
	Rev: AGCACGGAGACTCAACAACC	
*Pax3*	Fwd: AAACCCAAGCAGGTGACAAC	115
	Rev: AGACAGCGTCCTTGAGCAAT	
*Pax7*	Fwd: ATTACCTGGCCAAAAACGTG	105
	Rev: AGTAGGCTTGTCCCGTTTCC	
*Rplp0*	Fwd: GATTCGGGATATGCTGTTGG	108
	Rev: TCGGGTCCTAGACCAGTGTT	
*Six1*	Fwd: CCTGGGGCAAAATGATGTAT	112
	Rev: CAAAGCATGAGCAAGCCAAC	
*Six4*	Fwd: GGCCAGAGGTTGTTGTTTGT	109
	Rev: GGCAGCCAAGCTGTGTAAGT	
*Sox10*	Fwd: TACCTTTGCCTTGCACCCTT	111
	Rev: AAAGGGGCAGCGATGTGTTA	
*Trpm3*	Fwd: AAGGCTTTGACTTTCTGTCATCTG	105
	Rev: TTCAACAGTGGGTCCAATAGCA	
*Uba52*	Fwd: ATTGAGCCATCCCTTCGTCAG	111
	Rev: CTTCTTCTTGCGGCAGTTGAC	
*Ucp1*	Fwd: GGAGGTGTGGCAGTGTTCAT	112
	Rev: AAGCATTGTAGGTCCCCGTG	

### Microarrays

All microarray reagents, including the 36 Gene-Chips, and the instrumentation used for the microarray analyses were from Affymetrix (ThermoFischer Scientific Waltham, MA, USA). 250 ng total RNA were used for reverse transcription and the resulting cDNA was fragmented and labeled via the GeneChip^®^ WT PLUS Reagent Kit as per the manufacturer’s instructions (Affymetrix). The labeled cDNA samples were hybridized to Affymetrix MoGene 2.0 ST arrays and incubated in Genechip Hybridization Oven-645 (Affymetrix) rotating at 60 rpm at 45°C for 16 h. Subsequently, arrays were washed on a Genechip Fluidics Station-450 (Affymetrix) and stained with the Affymetrix HWS kit according to the manufacturer’s protocol. Finally, the chips were scanned with an Affymetrix Gene-Chip Scanner-3000-7G and the Affymetrix GCOS software was used for the generation of .dat and .cel files. Microarray data are available in the ArrayExpress database (www.ebi.ac.uk/arrayexpress) under the accession number E-MTAB-5755.

### Statistical analysis

The .cel files obtained by the microarray analyses were subjected to background correction, summarization and normalization by Robust Multiarray Analysis (RMA) and used for generation of .chp summarization files via the Expression Console^™^ Software 1.4 (Affymetrix), and subsequently were used to produce a three dimensional PCA plot. The .chp files were used for gene level differential expression quantification, accompanied by One-Way Between-Subject ANOVA statistical analysis via the Transcriptome Analysis Console 3.0 (Affymetrix). Transcripts having a p-value ≤ 0.05 and a linear FC ≥ ± 2 for comparison of E18.5 vs. E14.5 sample groups, or a FC ≥ ± 1.5 for E14.5 vs. E14.5 and E18.5 vs. E18.5 sample groups, were considered as differentially expressed genes (DEGs). Volcano plots were generated using the Transcriptome Analysis Console 3.0 (Affymetrix).

GraphPad Prism version 4.00 (GraphPad Software, La Jolla California USA, www.graphpad.com) was utilized for the statistical analysis of all qRT-PCR data. Unpaired t-test analyses were done when comparing the relative expression levels of one test group versus one control and one-way ANOVA followed by Bonferroni’s multiple comparisons test was performed when comparing multiple groups.

### Enrichment analyses

Gene enrichment analyses for DEGs identified upon the comparisons of different groups were performed with the databases *Gene Ontology for Biological Process* (GO BP) and *Cellular Component* (GO CC), as well as with *Wiki Pathways* (WP) using the *Enrichr* online enrichment tool [31]. A p-value ranking was applied to all enrichment analyses.

### Heatmaps and hierarchical clustering

Heatmaps and hierarchical clustering analyses were performed via the *ClustVis* online tool [32] using unit variance row scaling. Hierarchical average linkage clustering measuring the average Euclidean distance was applied for both rows and columns.

### Analysis of Ca_v_1.1 full length and Δ29 splice variants

To determine, within the same sample, the relative amount of Ca_v_1.1 transcripts containing or missing exon 29, i.e. Ca_v_1.1 full length and Δ29 respectively, the cDNA produced from 10 ng total RNA from each sample was used as template for PCR analysis. The region between Ca_v_1.1 exons 28 and 30 was amplified using the forward primer 5’-TCCTAATCGTCATCGGCAGC-3’ and the reverse primer 5’-TTTATCTGCGTCCCGTCCAC-3’. PCRs were performed using the DreamTaq Polymerase (Cat. #EP0703, ThermoFisher Scientific, Hagen, Germany) according to the manufacturer’s protocol. The PCR program consisted of an initial DNA denaturing step at 95°C for 3 minutes, followed by 35 cycles of 95°C for 30 seconds, 55°C for 30 seconds and 72°C for 1 minute; with a subsequent 5 minute elongation step at 72°C and a final holding step at 4°C. Transcripts containing exon 29 produced a 343 bp PCR product while those lacking exon29 resulted in a smaller product, 286 bp. The two PCR products were separated electrophoretically on 2% agarose gels and the bands were digitized via the INTAS documentation system (version 3.28.16.01.2009). Band intensities were quantified with the image analysis module implemented in the FluoView1000 software (Olympus, Japan). In the process of band intensity quantification, background correction was performed locally for each lane. Subsequently, the intensity integral of each band was calculated by summing the intensity values of all pixels belonging to that band. The sum of the two intensity integrals was regarded as 100%, so that the fractional intensity (in %) of each band, with or without exon 29, could be calculated.

## Results

### Altered gross morphology of RYR1^-/-^ and Ca_v_1.1^-/-^ fetuses at E18.5 but not at E14.5

**Fig 1:**
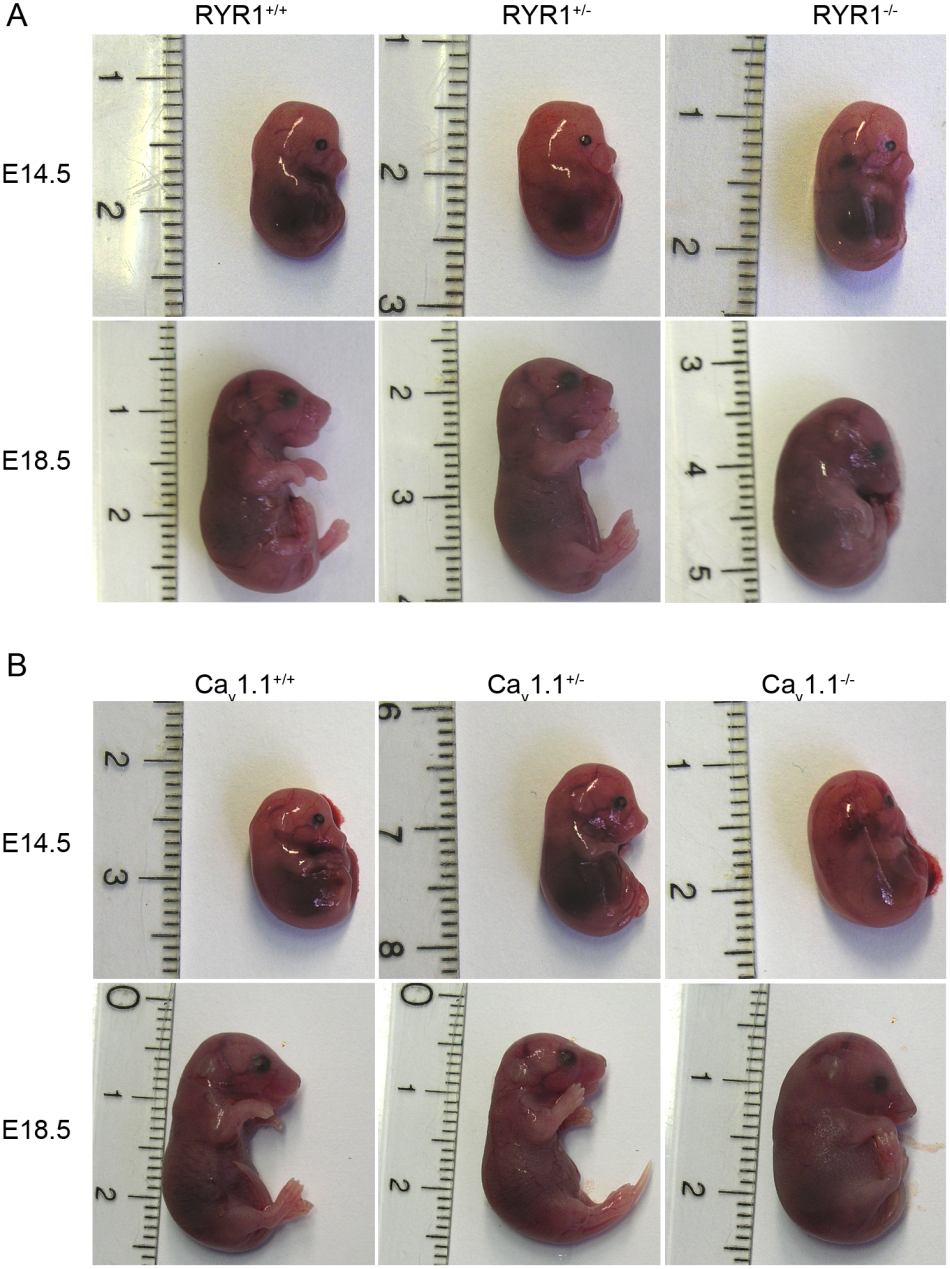
Gross fetal morphology at E14.5 and E18.5. Photographs of whole fetuses were obtained at E14.5 and E18.5 for RYR1^+/+^ (WT), RYR1^+/-^ and RYR1^-/-^ littermates (A), as well as for Ca_v_1.1^+/+^ (WT), Ca_v_1.1^+/-^ and Ca_v_1.1^-/-^ littermates (B).

First, we examined the effects of the absence of either RYR1 or Ca_v_1.1 on the gross morphological appearance at embryonic days E14.5 and E18.5. For this assessment 3 littermates from each genotype, RYR1^+/+^ (WT), RYR1^+/-^ and RYR1^-/-^, as well as Ca_v_1.1^+/+^ (WT), Ca_v_1.1^+/-^ and Ca_v_1.1^-/-^, at both E14.5 and E18.5, were used for whole embryos preparations (Fig 1). At E14.5 no apparent macroscopic differences in the morphology were observed between the WT, heterozygous (^+/-^) and homozygous (^-/-^) mutants of either mouse line. For the E18.5 stage, it was already known from previous studies that homozygous RYR1^-/-^ and Ca_v_1.1^-/-^ mutants, in comparison to their WT littermates, exhibit clear morphological alterations comprising a characteristic spinal curvature, smaller limbs and enlarged necks, as well as a smaller body size [24,27]. Our own observations on E18.5 mice confirm these findings, but show additionally that there is no distinguishable gross morphology between RYR1^+/-^ or Ca_v_1.1^+/-^ heterozygous fetuses compared to their WT littermates at this later stage (Fig 1).

### Altered morphology of homozygous, RYR1^-/-^ and Ca_v_1.1^-/-^, but also of heterozygous Ca_v_1.1^+/-^ fetuses at E14.5 and E18.5

**Fig 2:**
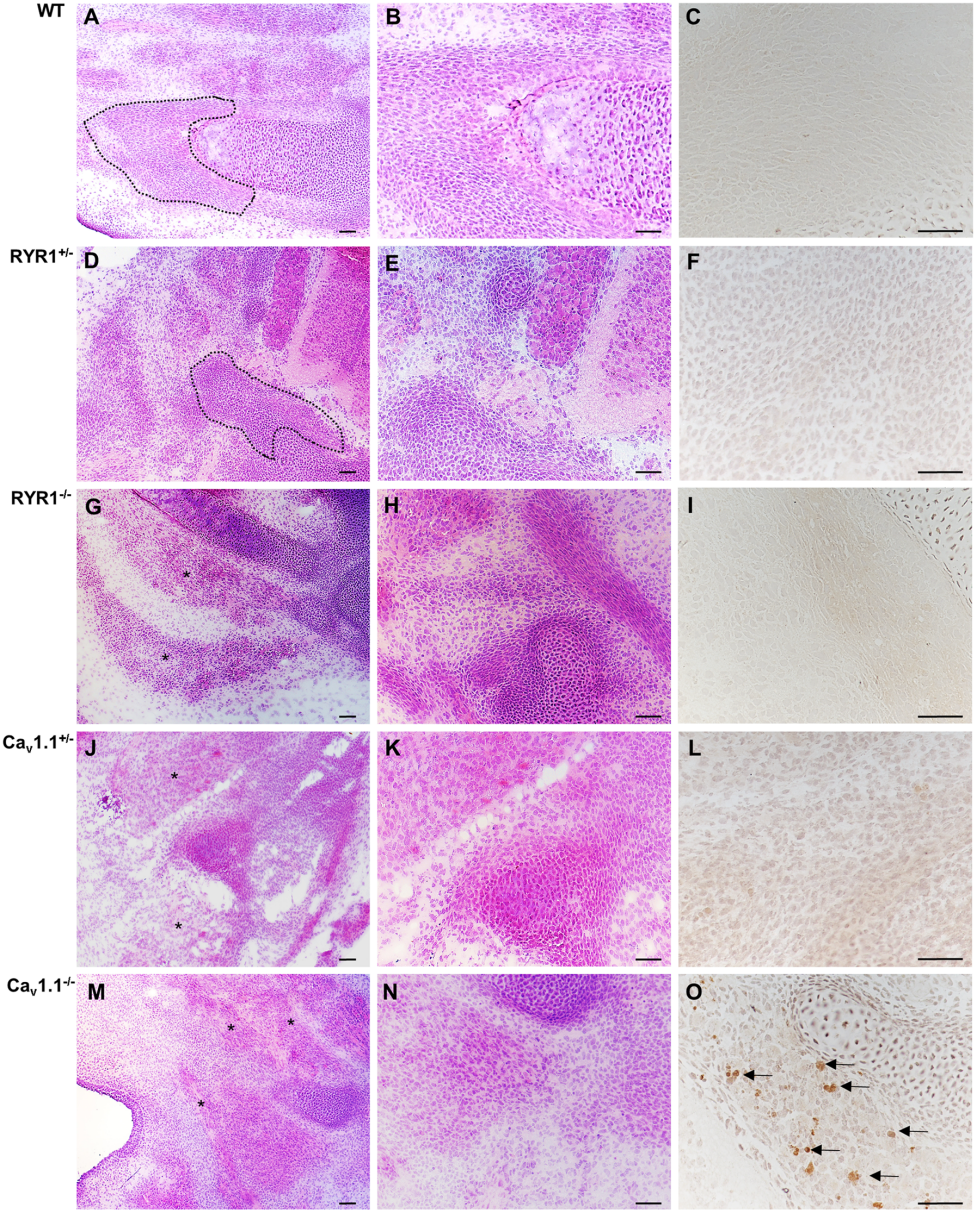
Histology of mouse limb skeletal muscle at embryonic day E14.5. Cross sections of the lower hind limb of a WT fetus (A-C), a RYR1^+/-^ fetus (D-F), a RYR1^-/-^ fetus (G-I), a Ca_v_1.1^+/-^ fetus (J-L), and a Ca_v_1.1^-/-^ fetus (M-O), respectively. At E14.5, the skeletal muscle of the hind limb of a WT fetus (A, B) as well as of a RYR1^+/-^ fetus (D, E) already harbor muscle fascicles (surrounded by dotted line) consisting of numerous muscle fibers while myoblasts were virtually absent. In contrast, the skeletal muscle of the hind limb of a RYR1^-/-^ (G, H), a Ca_v_1.1^+/-^ (J, K), and a Ca_v_1.1^-/-^ (M, N) fetus, respectively, exhibits disorganization (asterisks) or complete absence of muscle fascicles and numerous myoblasts. Immunohistochemistry with anti-activated caspase-3 reveals prominent apoptosis only in nuclei of the myotubes of a Ca_v_1.1^-/-^ fetus at E14.5 (O, arrows). H&E staining (A, B, D, E, G, H, J, K, M, and N); original magnification x100 (A, D, G, J, M) and x200 (B, E, H, K, N). Immunohistochemistry with rabbit anti-mouse activated caspase-3 (clone C92-605; BD Biosciences) and slight counterstaining with hemalum; original magnification x400. Scale bars correspond to 100 μm in all microphotographs.

**Fig 3:**
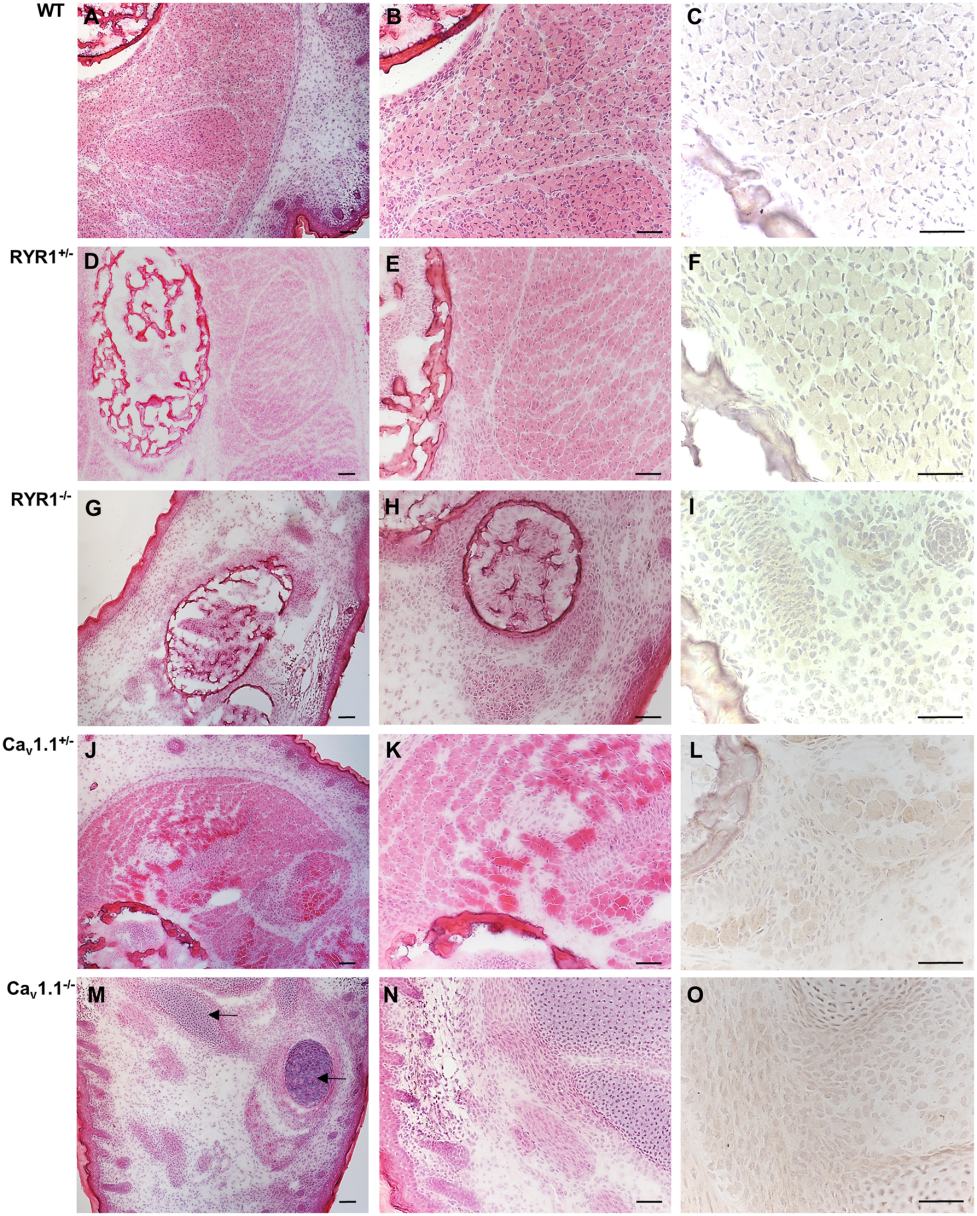
Histology of mouse limb skeletal muscle at embryonic day E18.5. Cross sections of the lower hind limb of a WT fetus (A-C), a RYR1^+/-^ fetus (D-F), a RYR1^-/-^ fetus (G-I), a Ca_v_1.1^+/-^ fetus (J-L), and a Ca_v_1.1^-/-^ fetus (M-O), respectively. At E18.5, the fetal skeletal muscles of the various genetically modified mice exhibit more pronounced morphological alterations. At this time point, the skeletal muscle of a WT fetus (A-C) is mature with regularly developed muscle fascicles consisting of normal sized muscle fibers as well as inconspicuous bone having reached a normal state of mineralization. In a RYR1^+/-^ fetus (D-F), skeletal muscle and bone are normally developed, thus, being similar to WT mice. In contrast, the skeletal muscle of a RYR1^-/-^ (G,H) and a Ca_v_1.1^-/-^ (M,N) fetus, respectively, consists predominantly of small, unorganized myotubes with lack of a fascicular organization. In addition, bone of the hind limb of a Ca_v_1.1^-/-^ (M-O) fetus is impaired in development as evidenced by persisting hyaline cartilage while mineralization has not been initiated (arrows in M). At day E18.5 apoptosis is completely absent from all mutant strains as evidenced by the absence of nuclear immunoreaction in immunohistochemistry with anti-activated caspase-3. H&E staining (A, B, D, E, G, H, J, K, M, and N); original magnification x100 (A, D, G, J, M) and x200 (B, E, H, K, N). Immunohistochemistry with rabbit anti-mouse activated caspase-3 (clone C92-605; BD Biosciences) and slight counterstaining with hemalum; original magnification x400. Scale bars correspond to 100 μm in all microphotographs.

Next, we analyzed serial cross sections from the hind limbs of WT, heterozygous (^+/-^), and homozygous (^-/-^) RYR1 and Ca_v_1.1 mice at E14.5 (Fig 2) and E18.5 (Fig 3). At E14.5 WT muscles consisted predominantly of myotubes; some primary muscle fibers were already detectable and fascicle formation was already initiated (Fig 2A and 2B). While the morphology of heterozygous RYR1^+/-^ animals (Fig 2D and 2E) was similar to WT fetuses, the hind limb muscles of homozygous RYR1^-/-^ mutants (Fig 2G and 2H) were predominated by myotubes with only single muscle fibers of a decreased fiber caliber. In addition, there was no evidence for any organization of muscle fascicles in RYR1^-/-^. At E14.5, morphological alterations in muscles obtained fromCa_v_1.1^-/-^ animals (Fig 2M and 2N) were similar to that of RYR1^-/-^ mutants. However, in contrast to heterozygous RYR1^+/-^ animals which were morphologically similar to the WT fetuses, the disorganization of muscle fascicles obtained from heterozygous Ca_v_1.1^+/-^ animals (Fig 2J and 2K) was similar to those of homozygous Ca_v_1.1^-/-^ mice (Fig 2M and 2N). Thus, homozygous Ca_v_1.1^-/-^ state displayed the most severe phenotype, with skeletal muscles consisting almost exclusively of small caliber myotubes and myoblasts while mature muscle fibers were virtually absent (Fig 2M and 2N). Apoptosis of a small fraction of myotubes has only been identified in the skeletal muscles of E14.5 Ca_v_1.1^-/-^ fetuses as evidenced by nuclear anti-activated caspase-3 staining (Fig 2O, arrows) while it was absent in the other fetuses.

At E18.5, WT and RYR1^+/-^ muscles were normally developed and consisted predominantly of well-differentiated muscle fibers organized in fascicles (Fig 3A–3F), thus, being in line with our own recent study [28]. At this time point, the skeletal muscle of RYR1^-/-^ fetuses consisted predominantly of myotubes and small, disorganized fibers accompanied by a severely affected fascicle formation, hinting a developmental retardation (Fig 3G and 3H). In contrast, the skeletal muscles of both heterozygous Ca_v_1.1^+/-^ fetuses (Fig 3J and 3K) and homozygous E18.5 Ca_v_1.1^-/-^ fetuses (Fig 3M and 3N) still exhibited signs of immaturity as characterized by a predominance of myoblasts and myotubes and only a fraction of muscle fibers showing evidence of a beginning organization into fascicles in heterozygous Ca_v_1.1^+/-^ fetuses (Fig 3J and 3K). In contrast, the skeletal muscle of homozygous E18.5 Ca_v_1.1^-/-^ fetuses (Fig 3M and 3N) completely persisted in immature state. In addition, maturation of bone of the hind limbs of homozygous E18.5 Ca_v_1.1^-/-^ fetuses (Fig 3; arrows in M) was markedly retarded with persistence of hyaline cartilage at a time point when mineralization of bones should be active.

### Only discrete changes were observed in the expression of myogenic regulatory factors (MRFs) in RYR1^-/-^ and Ca_v_1.1^-/-^ skeletal muscle at E14.5 and E18.5

**Fig 4:**
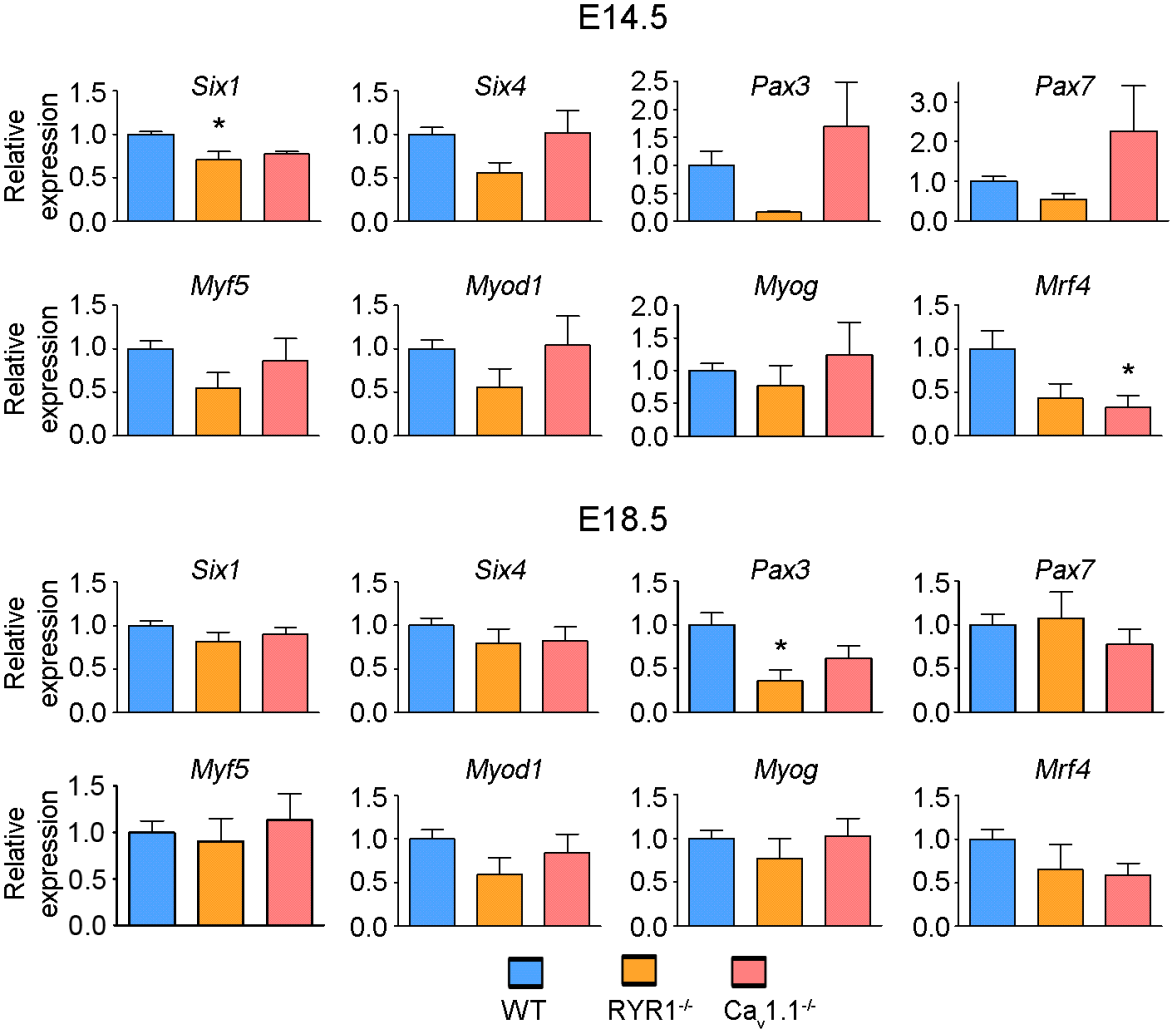
Comparison of the expression of MRFs in skeletal muscle from WT, RYR1^-/-^ and Ca_v_1.1^-/-^ mice at E14.5 and at E18.5. Relative expression levels of *Six1*, *Six4*, *Pax3*, *Pax7*, *Myf5*, *Myod1*, *Myog* and *Mrf4* in WT, RYR1^-/-^ and Ca_v_1.1^-/-^ samples (each n = 6) at E14.5 (upper part) and E18.5 (lower part) were obtained by qRT-PCR analyses, using *Cytb* as endogenous control. Expression levels of WT samples were set to 1. One way ANOVA with Bonferroni’s Multiple Comparison tests were performed for each gene, *represents a p-value ≤ 0.05. Error bars are S.E.M.

The genes *Six1*, *Six4*, *Pax3*, *Pax7*, *Myf5*, *Myod1*, *Myog* and *Mrf4* encode canonical MRFs that affect the expression of multiple genes throughout skeletal muscle development [10]. We previously reported changes in the expression levels of several MRFs at E18.5 in RYR1^-/-^ skeletal muscle [28]. In the present study, we investigated MRFs expression in limb skeletal muscle at E14.5 and E18.5. This was done via qRT-PCRs of samples from 6 RYR1^-/-^, 6 Ca_v_1.1^-/-^ and 6 WT animals (Fig 4). At E14.5, a slight but significant downregulation of *Six1* (0.7-fold of WT) was observed in RYR1^-/-^ samples and a stronger downregulation of *Mrf4* (0.33-fold of WT)–in the Ca_v_1.1^-/-^ samples with no significant changes in the other MRFs in both lines. However, in the RYR1^-/-^ samples *Pax3* exhibited a tendency towards downregulation, which was significant at E18.5. No further statistically significant changes in the expression of any of the MRFs were found for the RYR1^-/-^ and Ca_v_1.1^-/-^ samples at E18.5.

### Attenuated Ca_v_1.1 isoform-switch in RYR1^-/-^ limb skeletal muscle at E14.5

**Fig 5:**
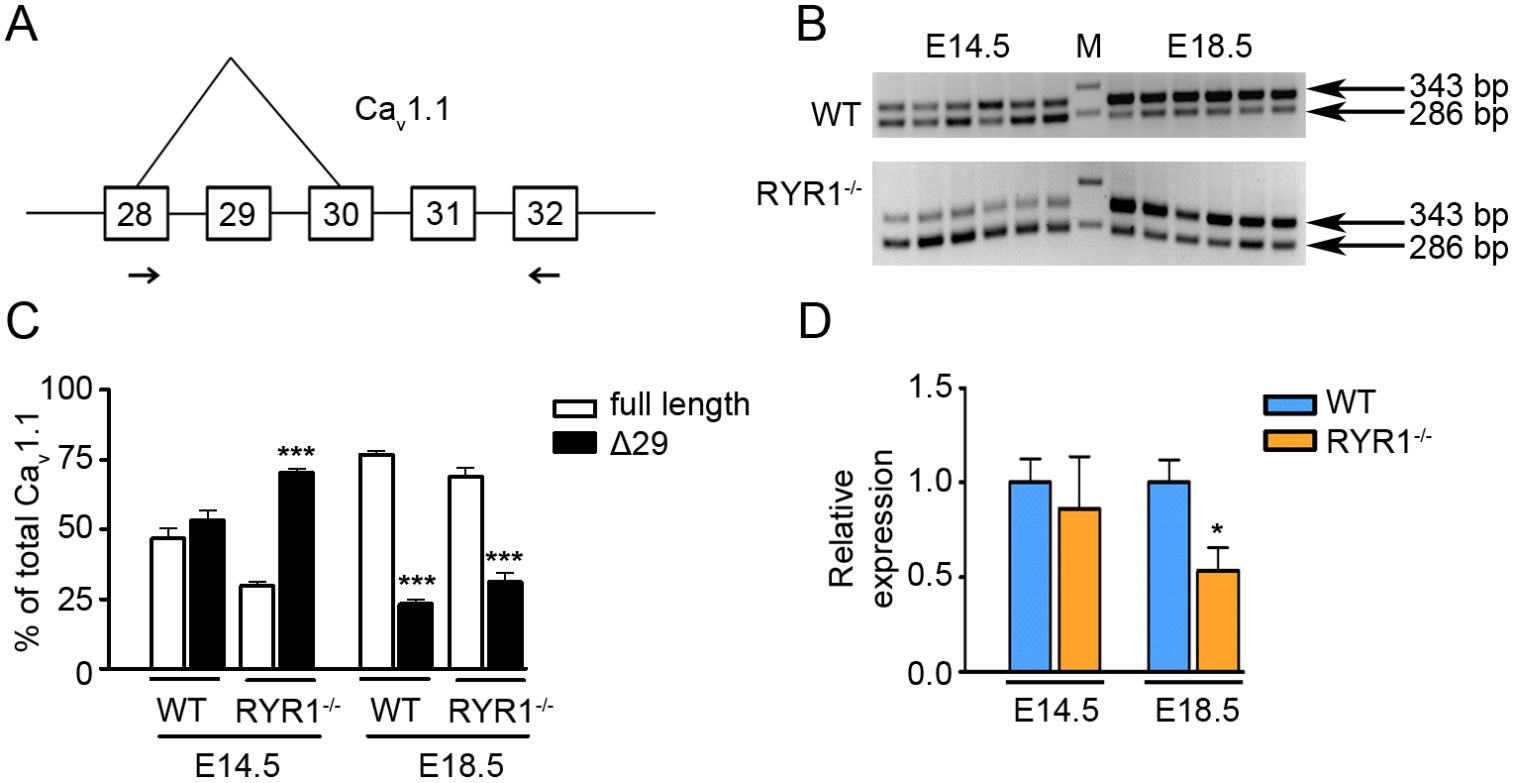
Ca_v_1.1 splice variants in WT and RYR1^-/-^ skeletal muscle. (A) Graphical representation of the genomic exon 29 vicinity of murine full-length and Δ29 Ca_v_1.1 (NCBI Reference Sequence: NM_001081023.1) splice variants. Arrows indicate the primer binding positions used for amplification of exons 28–32. (B) PCR products of the full-length (343 bp) and Δ29 (286 bp) Ca_v_1.1 splice variants. (C) Full-length (343 bp) and Δ29 (286 bp) splice variants as percentage of total Ca_v_1.1 mRNA in limb skeletal muscle from WT and RYR1^-/-^ animals at E14.5 and E18.5. (D) Relative expression of total Ca_v_1.1 mRNA measured via qRT-PCR in RYR1^-/-^ vs. WT skeletal muscle at E14.5 and E18.5, using *Cytb* as endogenous control. *t*-tests were performed for comparison of Δ29 vs. full-length splice variants (C) and for WT vs. RYR1^-/-^ (D) in each group; * indicates *p* values <0.05 and *** *p* values < 0.001; error bars are S.E.M.

It has been previously reported that a splice variant of Ca_v_1.1 lacking exon 29 (Ca_v_1.1 Δ29) is highly expressed in skeletal muscle during embryonic development and that its expression levels diminish at birth and almost disappear until the third week of postnatal development [33,34]. Unlike the full-length Ca_v_1.1, which predominates in fully differentiated fibers and which only weakly conducts Ca^2+^ currents, the Δ29 variant is characterized by a much higher Ca^2+^ conductance and has been implicated in patterning of the neuromuscular junction during development [34]. To investigate whether the absence of RYR1 has an impact on the normal developmental pattern of Ca_v_1.1 splice variants, we analyzed the relative expressions levels of the two Ca_v_1.1 splice forms (full length and Δ29) in WT and RYR1^-/-^ limb skeletal muscle. The region between exons 28 and 32 of the Ca_v_1.1 transcript was amplified via PCR (Fig 5A), using cDNA from the limb skeletal muscles of 6 WT and 6 RYR1^-/-^ animals at E14.5 and E18.5. The full length Ca_v_1.1 transcript yielded a 343 bp PCR product and the Δ29 Ca_v_1.1 transcript—a 286 bp PCR product. The PCR products were subjected to agarose gel electrophoresis (Fig 5B, S1 Fig), the intensities of the bands were measured and used for calculation of the relative amount of each splice variant as a percentage of the total Ca_v_1.1 transcript (Fig 5C). At E14.5 each of the splice variants constituted approximately 50% of the total Ca_v_1.1 transcript in the WT samples, whereas in the RYR1^-/-^ samples the Δ29 Ca_v_1.1 variant amounted for 70% of the total Ca_v_1.1 transcript. At E18.5 the transcript levels for the full length Ca_v_1.1 were significantly higher than those for Δ29 Ca_v_1.1, in both WT and RYR1^-/-^ (77% in WT and 69% in RYR1^-/-^). However, qRT-PCR revealed an approximately 2-fold lower total level of Ca_v_1.1 mRNA in RYR1^-/-^ compared to WT limb skeletal muscle at E18.5. These results are in agreement with previous studies demonstrating a 2-fold reduced Ca_v_1.1 protein expression, as well as a strong decrease of L-type Ca2+ current density and charge movements in skeletal muscle from RYR1^-/-^ neonates [29,35,36]. The prolonged prevalence of Ca_v_1.1 Δ29, as well as a reduced Ca_v_1.1 expression in the absence of RYR1 is indicative of an impaired skeletal muscle development. These results also infer possible defects in proper neuromuscular junction formation that might have various downstream effects on the myogenic program.

### Global transcriptome analyses reveal distinct profiles of RYR1^-/-^ and Ca_v_1.1^-/-^ limb skeletal muscle at E18.5

**Fig 6:**
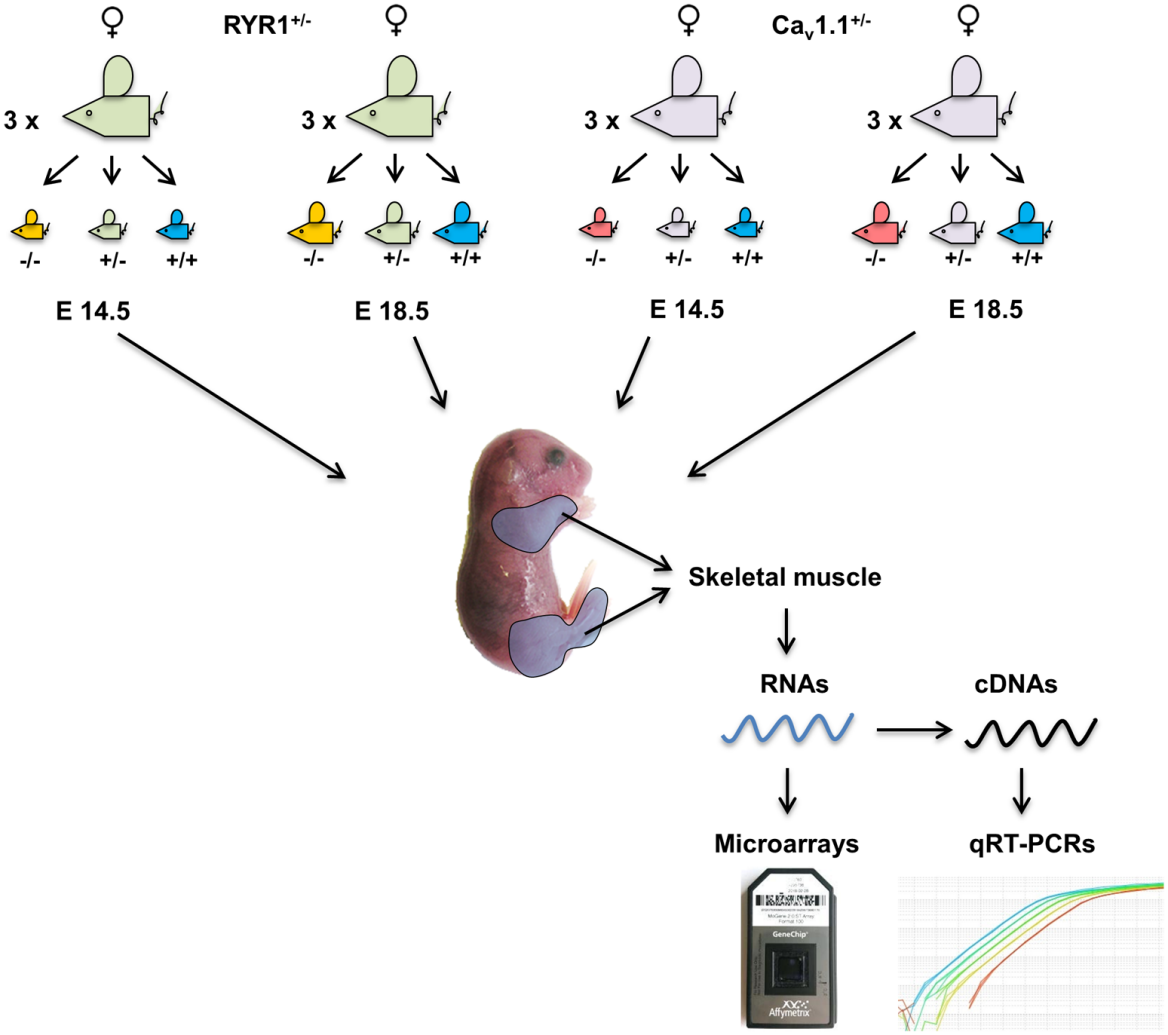
Schematic representation of the samples used in the present study and work flow for the subsequent gene expression analyses. Heterozygous Ca_v_1.1^+/-^ and RYR1^+/-^ male and female animals were subjected to timed pairings. At E14.5 and E18.5 post coitum three pregnant females of each line were sacrificed and skeletal muscle samples were collected from the front and hind limbs of 3 littermates (n = 3) of each of the genotypes—WT, heterozygous (Ca_v_1.1^+/-^ or RYR1^+/-^) and homozygous (Ca_v_1.1^-/-^ or RYR1^-/-^) mutants. Samples were handled separately and used for total RNA extractions and subsequent MA and qRT-PCR analyses.

In order to elucidate the global changes in gene expression that accompany secondary myogenesis in mouse limb skeletal muscle from E14.5 to E18.5, we performed microarray analyses (MAs). In particular, at each time point (E14.5 and E18.5) the skeletal muscles from the front and hind limbs of 3 littermates of each of the genotypes RYR1^+/+^ (WT), RYR1^+/-^ and RYR1^-/-^, as well as Ca_v_1.1^+/+^ (WT), Ca_v_1.1^+/-^ and Ca_v_1.1^-/-^ were collected and used for total RNA extraction (Fig 6). After evaluation of their quality (S2 Fig), the RNAs were subjected to MAs, with each MA covering 41,345 probes.

**Fig 7:**
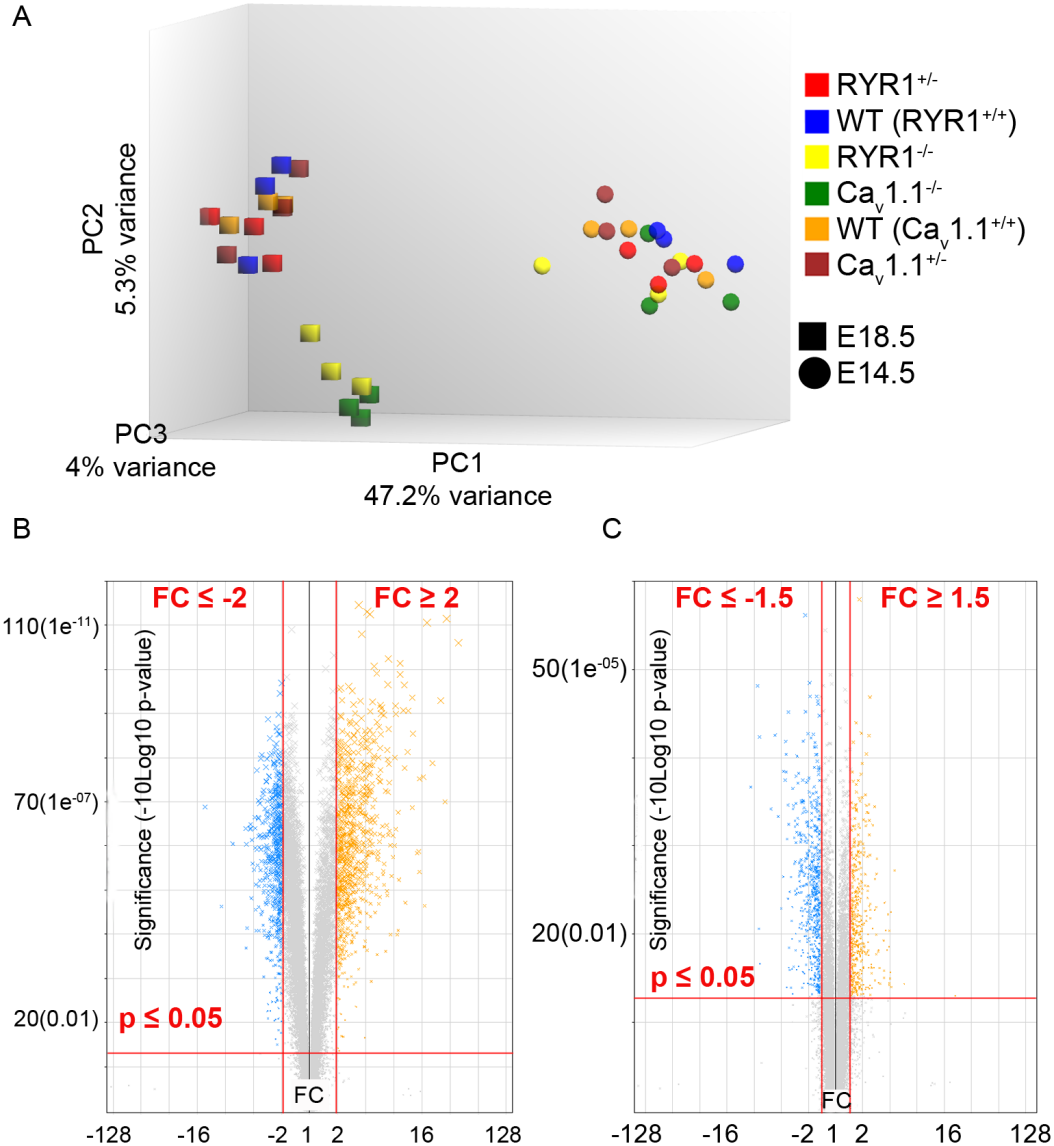
Initial MAs and DEGs analysis. (A) A principal component analysis (PCA) was performed for all samples with all their genes detected by the MAs via the Transcriptome Analysis Console 3.0 (Affymetrix^®^). (B) When comparing different developmental stages (E18.5 vs. E14.5), the cut-off criteria for being considered as DEG were an FC ≥ +2 or ≤-2, and a p ≤ 0.05 (the example shown is from the comparison WT E18.5 vs. WT E14.5). (C) When comparing groups from the same developmental stage, the cut-off criteria were an FC ≥ +1.5 or ≤-1.5, and a p ≤ 0.05 (the example shown is from the comparison Ca_v_1.1^-/-^ E18.5 vs. WT E18.5).

In order to analyze whether the different genotypes, developmental stages, and biological replicates from the same genotype and stage, segregate into distinct groups on the basis of their variance in expression, a principal component analysis (PCA) was performed for all genes identified in the MAs. As Fig 7A shows, the most prominent separation is that between developmental stages (PC 1 = 47.2% variance). Only secondary is the separation within the E18.5 group (squares in Fig 7A) between homozygous (Ca_v_1.1^-/-^ or RYR1^-/-^) mutants and the other genotypes (PC 2 = 5.3% variance), which is most distinct for the Ca_v_1.1^-/-^ genotype (green squares in Fig 7A). In contrast, PCA revealed no clear separation of genotypes for the E14.5 stage. Thus, the PCA analysis demonstrates that the E18.5 limb skeletal muscle transcriptomes of the homozygous mutants share more similarity with each other than with any other analyzed genotype.

Our MAs focus on two aspects, first a developmental aspect, by performing comparisons of the same genotype for the two stages E14.5 and E18.5, as well as a genotype aspect, by performing comparisons within the same embryonic day but for distinct genotypes. In the first case we considered a gene as being differentially regulated between the two developmental groups when its p-value was ≤ 0.05 and when its linear FC was ≤-2 or ≥+2 (Fig 7B). In the other case (focus on genotype-specific changes) our criteria for classifying a gene as a DEG were an FC of ≤-1.5 or ≥+1.5, and a p≤0.05 (Fig 7C). Only DEGs meeting these criteria (Table 2, S1 Table) were subjected to further analysis. Furthermore, the samples from heterozygous (RYR1^+/-^; Ca_v_1.1^+/-^) animals revealed only a handful of DEGs when compared to their WT littermates, at both E14.5 and E18.5. Therefore our further analysis focuses on the comparison of transcriptomic changes in homozygous mutant (RYR1^-/-^; Ca_v_1.1^-/-^) vs. WT limb skeletal muscle.

**Table 2:** Differentially regulated genes for various comparisons of genotypes.

Test group	Comparisson(Test vs. Control group)	Total DEGs	Downregulated DEGs	Upregulated DEGs
WT	E18.5 vs. WT E14.5	1314	541	773
RYR1^+/-^	E18.5 vs. RYR1^+/-^ E14.5	1426	611	815
	E14.5 vs. WT E14.5	36	27	9
	E18.5 vs. WT E18.5	21	13	8
RYR1^-/-^	E18.5 vs. RYR1^-/-^ E14.5	812	311	501
	E14.5 vs. WT E14.5	61	32	29
	E18.5 vs. WT E18.5	493	304	189
Ca_v_1.1^+/-^	E18.5 vs. Ca_v_1.1^+/-^ E14.5	1079	433	646
	E14.5 vs. WT E14.5	8	5	3
	E18.5 vs. WT E18.5	33	10	23
Ca_v_1.1^-/-^	E18.5 vs. Ca_v_1.1^-/-^ E14.5	900	282	618
	E14.5 vs. WT E14.5	97	66	31
	E18.5 vs. WT E18.5	1047	571	476

### Validation of the MAs via qRT-PCRs

**Fig 8:**
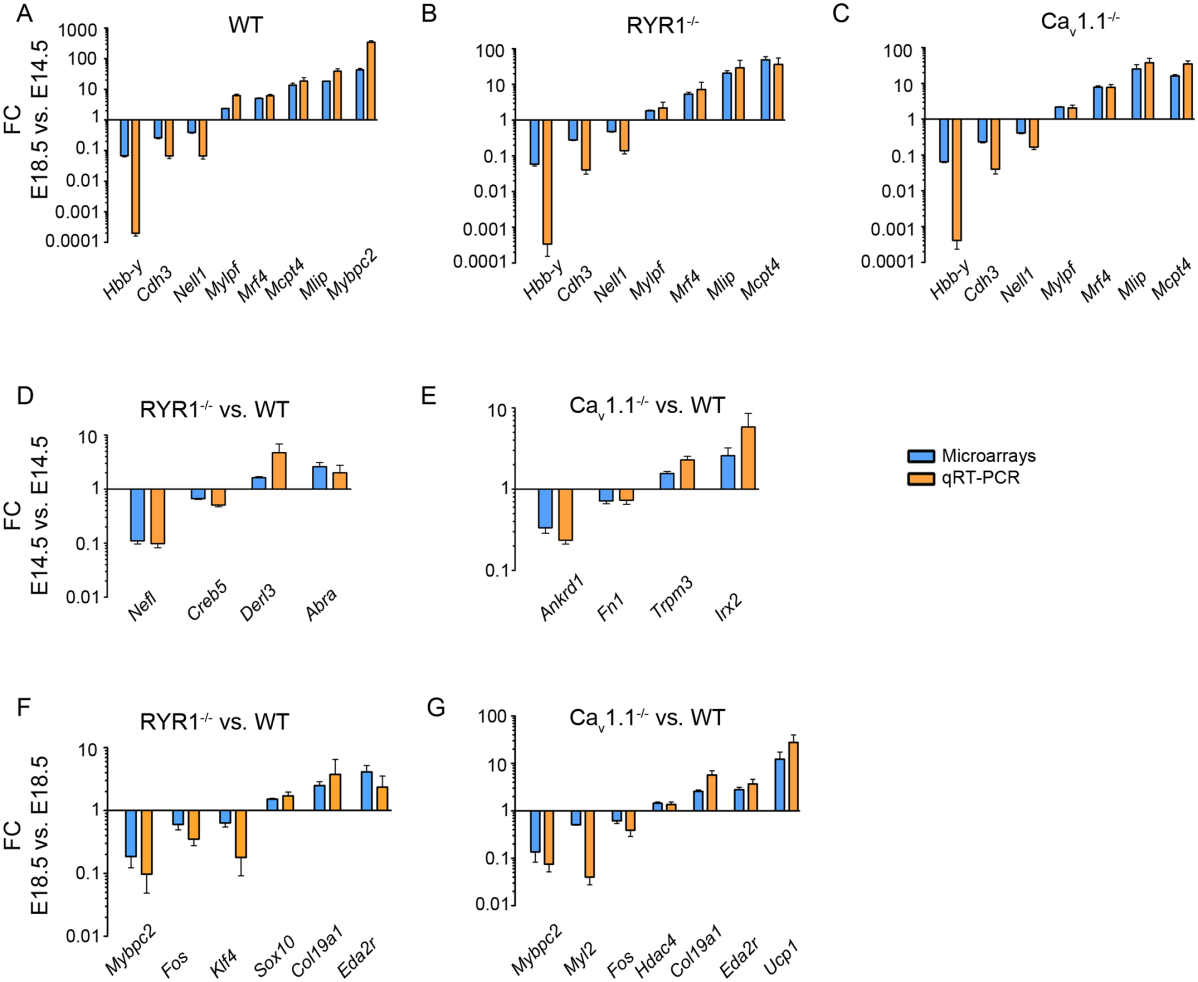
Validation of the MAs results via qRT-PCRs. (A-C) Validation of DEGs found in the comparison E18.5 vs. E14.5 for the same genotype. (A), WT vs. WT, 8 genes, n = 6 biological replicates per group; (B), RYR1^-/-^ vs. RYR1^-/-^, 7 genes, n = 3 biological replicates per group; (C), Ca_v_1.1^-/-^ vs. Ca_v_1.1^-/-^, 7 genes, n = 3 biological replicates per group. (D-E) Validation of selected genes found to be differentially regulated in E14.5 samples from RYR1^-/-^ muscle (D) and Ca_v_1.1^-/-^ muscle (E), when compared to E14.5 WT. (D & E), 4 genes for each genotype comparison, n = 3 biological replicates per group. (F-G) Validation of selected genes found to be differentially regulated in E18.5 samples from RYR1^-/-^ muscle (F) and Ca_v_1.1^-/-^ muscle (G), when compared to E18.5 WT. (F), 6 genes, n = 3 biological replicates per group; (G), 7 genes, n = 3 biological replicates per group. In all MA and qRT-PCR analyses the FCs of the control samples were set to 1. The relative expression levels obtained by qRT-PCR analysis were normalized to *Cytb*, which was used as endogenous control. Error bars are S.E.M.

Next, the results obtained using MA were validated via qRT-PCRs. For validation of the comparison of gene expression between E14.5 and E18.5, seven to eight down- or upregulated DEGs, covering a broad FC spectrum were randomly selected. Fig 8A–8C presents the results of this validation for WT, RYR1^-/-^, and Ca_v_1.1^-/-^ samples, respectively. Due to the eminently lower number of DEGs detected by the MAs in E14.5 vs. E14.5 comparisons, fewer genes were used for the validation of RYR1^-/-^ vs. WT and Ca_v_1.1^-/-^ vs. WT at this developmental stage (Fig 8D and 8E). Validation of the E18.5 vs. E18.5 comparisons comprised six (RYR1^-/-^ vs. WT, Fig 8F) and seven (Ca_v_1.1^-/-^ vs. WT, Fig 8G) genes, respectively. As Fig 8 shows, the direction of changes in expression detected by our MAs, and for many of the genes also the FC magnitude, was firmly recapitulated by our quantitative PCR analysis. Therefore, we conclude that our results obtained using MAs give a reliable picture of the changes in expression in the various skeletal muscle samples.

### Transcriptomes of RYR1^-/-^ and Ca_v_1.1^-/-^ limb skeletal muscle deviate from WT already at E14.5

**Fig 9:**
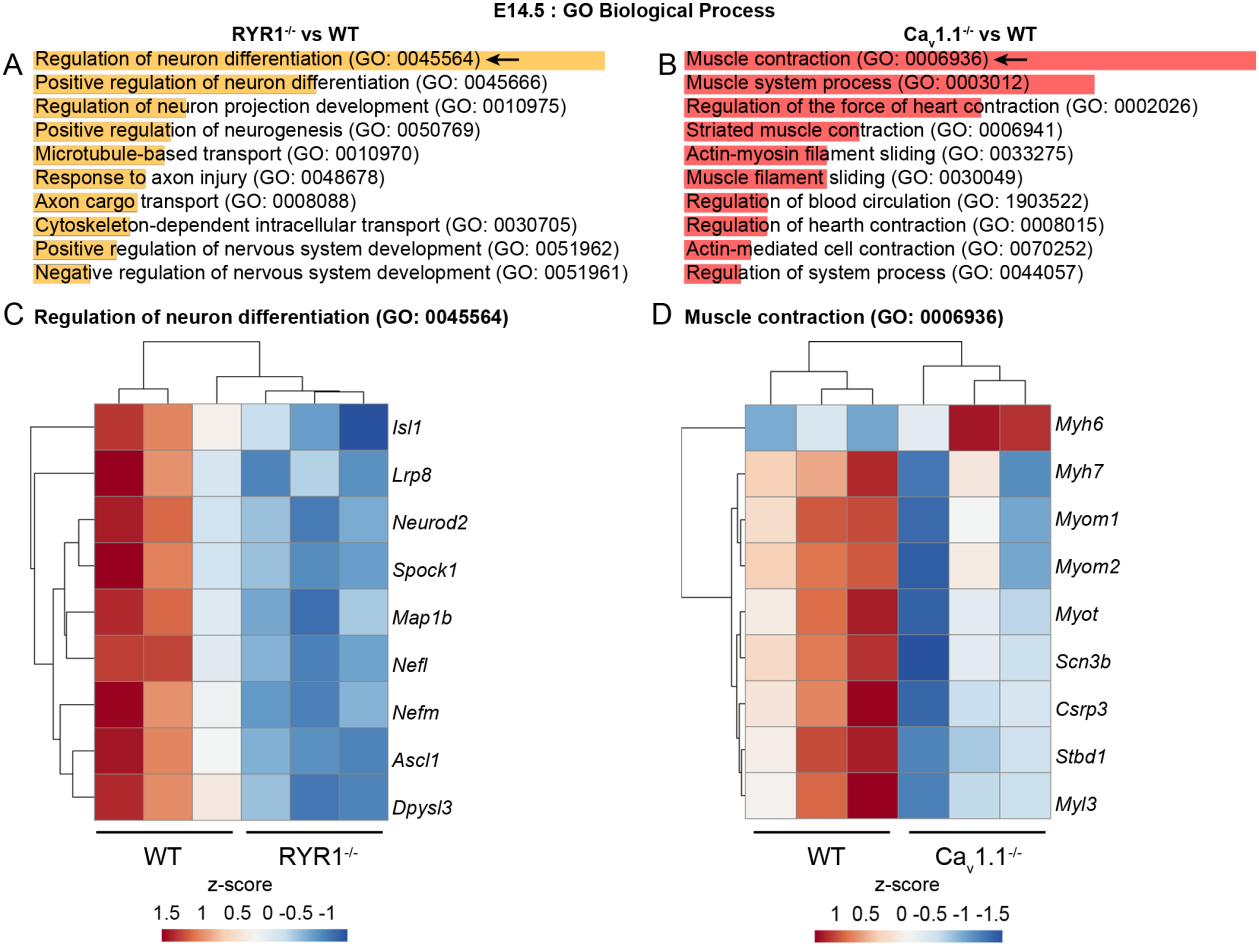
Biological processes affected by the RYR1^-/-^ and Ca_v_1.1^-/-^ mutations at E14.5. GO BP enrichment analyses were performed for the DEGs identified in the RYR1^-/-^ (A) and Ca_v_1.1^-/-^ (B) samples when compared to WT littermates samples at E14.5. The ten most significantly enriched categories for each analysis are shown. Arrows indicate categories presented as heat maps in (C) and (D). The enrichment analyses was performed via the Enrichr online tool [31], length of the bars represents the significance (p-value). Heatmaps were generated for the DEGs enriched in the “Regulation of neuron differentiation” biological process in RYR1^-/-^ samples (C) and for the DEGs enriched in “Muscle contraction” biological process in Ca_v_1.1^-/-^ samples (D). The heatmaps were generated from the MAs intensity levels of each gene via ClustVis [32]. Hierarchical average linkage clustering using the Euclidean distance was performed for all rows and columns.

An important question in our analysis was about the time of onset of transcriptomic alterations during skeletal myogenesis when either RYR1 or Ca_v_1.1 is absent. We therefore compared the E14.5 MA profiles of RYR1^-/-^ and Ca_v_1.1^-/-^ limb skeletal muscle to those of their WT littermates. 61 DEGs were identified in the RYR1^-/-^ samples and 97 DEGs in the Ca_v_1.1^-/-^ samples (Table 2, S1 Table). Interestingly, only two DEGs—the solute carrier family 44, member 5 (*Slc44a5*) and Der1-like domain family, member 3 (*Derl3*) were found in both RYR1^-/-^ and Ca_v_1.1^-/-^ samples, suggesting that the absence of RYR1 or Ca_v_1.1 at this early stage might affect distinct cellular processes. Indeed GO BP enrichment analysis revealed that processes related to innervation and to cellular transport were most significantly influenced in RYR1^-/-^ samples (Fig 9A, S2 Table), whereas the most affected processes in Ca_v_1.1^-/-^ samples were associated with muscle contraction (Fig 9B, S2 Table). Heatmaps were generated for the DEGs related to the most significantly altered processes, i.e., “Regulation of neuron differentiation” in RYR1^-/-^ samples (Fig 9C, S3 Table) and “Muscle contraction” in Ca_v_1.1^-/-^ samples (Fig 9D, S3 Table). Both heatmaps show a downregulation of all DEGs related to these two processes, with the only exception in Ca_v_1.1^-/-^ being *Myh6*, which encodes cardiac myosin heavy polypeptide 6, alpha.

### Substantial overlap of RYR1^-/-^ and Ca_v_1.1^-/-^ limb skeletal muscle transcriptomes at E18.5

**Fig 10:**
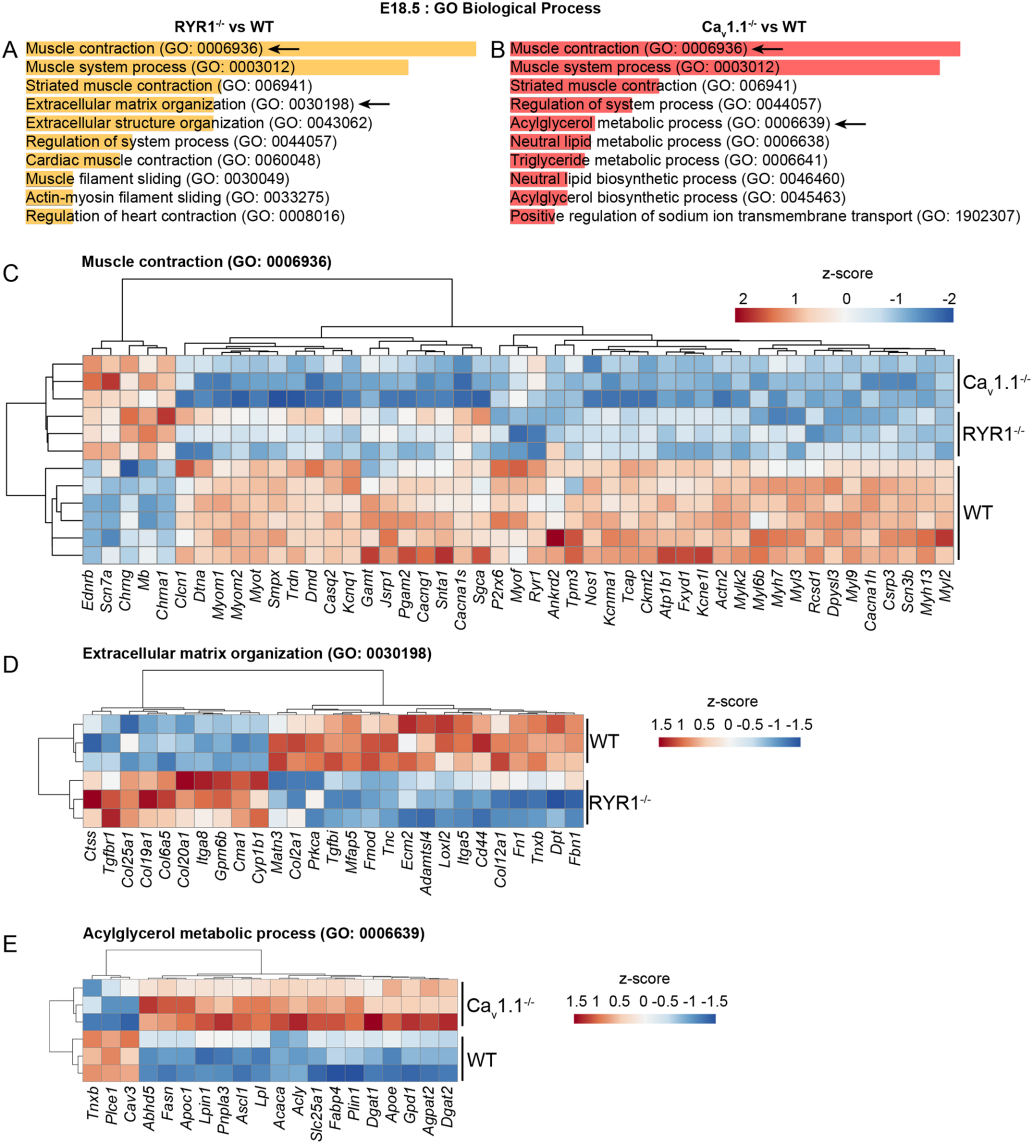
Biological processes affected by the RYR1^-/-^ and Ca_v_1.1^-/-^ mutations at E18.5. GO BP enrichment analyses were performed for the DEGs identified in the RYR1^-/-^ (A) and Ca_v_1.1^-/-^ (B) samples with their E18.5 WT littermates serving as reference. The ten most significantly enriched categories for each analysis are shown. Arrows indicate categories presented as heat maps in (C), (D) and (E). The enrichment analyses was performed via the Enrichr online tool [31], the length of the bars corresponds to the significance (p-value). Heatmaps were generated for the DEGs enriched in the “Muscle contraction” biological process in RYR1^-/-^ and Ca_v_1.1^-/-^ samples (C); for the DEGs enriched in “Extracellular matrix organization” biological process in RYR1^-/-^ samples (D); and for the DEGs enriched in “Acylglycerol metabolic process” biological process in Ca_v_1.1^-/-^ samples (E). Heatmaps were generated from the MAs intensity levels of each included gene via ClustVis [32]. Hierarchical average linkage clustering using the Euclidean distance was performed for all rows and columns.

To determine how the moderate changes found in the transcriptomes of RYR1^-/-^ and Ca_v_1.1^-/-^ at E14.5 evolve until the later stage of secondary myogenesis, we performed analogous screens using samples from E18.5. Using WT E18.5 as the reference, 493 DEGs were identified in the RYR1^-/-^ samples and 1,047 DEGs in the Ca_v_1.1^-/-^ samples (Table 2, S1 Table). 328 DEGs were shared by both RYR1^-/-^ and Ca_v_1.1^-/-^ samples, which, with respect to the total number of DEGs identified in the E18.5 comparisons, is a significant overlap (66.5% of all RYR1^-/-^ DEGs were identical to 31.3% of all Ca_v_1.1^-/-^ DEGs). These results reveal a substantial similarity in the transcriptomic profiles of RYR1^-/-^ and Ca_v_1.1^-/-^ limb skeletal muscle at the final stages of embryogenesis and of secondary myogenesis. Among the DEGs shared by both mutants at E18.5 were members of signaling pathways with critical roles in skeletal muscle development, like the MAPK, PI3K-AKT, Wnt, cAMP and cGMP-PKG pathways (S4 Table). Further analysis of all DEGs shared by RYR1^-/-^ and Ca_v_1.1^-/-^ at E18.5 using GO BP, demonstrated that the three most significantly affected processes were identical in both mutants and were all related to muscle contraction (Fig 10A and 10B, S5 Table). However, GO BP enrichment analysis also generated assignments of DEGs to processes which were distinct in RYR1^-/-^ and Ca_v_1.1^-/-^. For instance, highly enriched in the RYR1^-/-^ samples were genes related to extracellular matrix and structure organization, while a group of DEGs in Ca_v_1.1^-/-^ samples is related to fatty acid and lipid metabolism. To appreciate the direction in which these processes were altered in the two mutants, heatmaps were constructed for the DEGs involved in *Muscle contraction* (as stated above the most significantly enriched biological process for both groups; Fig 10C, S6 Table). Heat maps were also generated for “Extracellular matrix organization”, using the DEGs from RYR1^-/-^ samples (Fig 10D, S7 Table) and for “Acylglycerol metabolic process”, using the DEGs from Ca_v_1.1^-/-^ samples (Fig 9E, S8 Table). As the heat map in Fig 10C shows, a very large proportion of genes (44 of the 49 DEGs) assigned by GO BP to the process of “Muscle contraction” were downregulated in RYR1^-/-^, in Ca_v_1.1^-/-^, or in both mutants. Only 5 DEGs were positively regulated (Fig 10C). A significant fraction of the negatively regulated DEGs encode constituents of the sarcomere, like *Myl2*, *Myl3*, *Myl6b*, *Myl9*, *Myh3*, *Myh7*, *Csrp3*, *Tcap*, *Tpm3*, *Myom1* and *Myom2*, which explains the scarcity of myofibrils and probably also the abnormalities in sarcomere arrangement in limb skeletal muscle from RYR1^-/-^ and Ca_v_1.1^-/-^ mice at the perinatal stage [24,27].

17 out of the 27 DEGs related to “Extracellular matrix organization” were negatively regulated in RYR1^-/-^ compared to WT samples (Fig 10D). Among the 10 positively regulated genes four encoded collagens, hinting to potential changes in the composition of the ECM in RYR1^-/-^ limb skeletal muscle.

The vast majority, 17 out of 20, of the DEGs relating in the enrichment analysis to the “Acylglycerol metabolic process”, displayed a higher expression level in Ca_v_1.1^-/-^ muscle when compared to their WT littermates (Fig 10E). We therefore assume the presence of an enhanced lipid metabolism in limb skeletal muscle of E18.5 Ca_v_1.1^-/-^ mice.

### The transcriptome of WT and mutant skeletal muscle at E18.5 vs. E14.5

**Fig 11:**
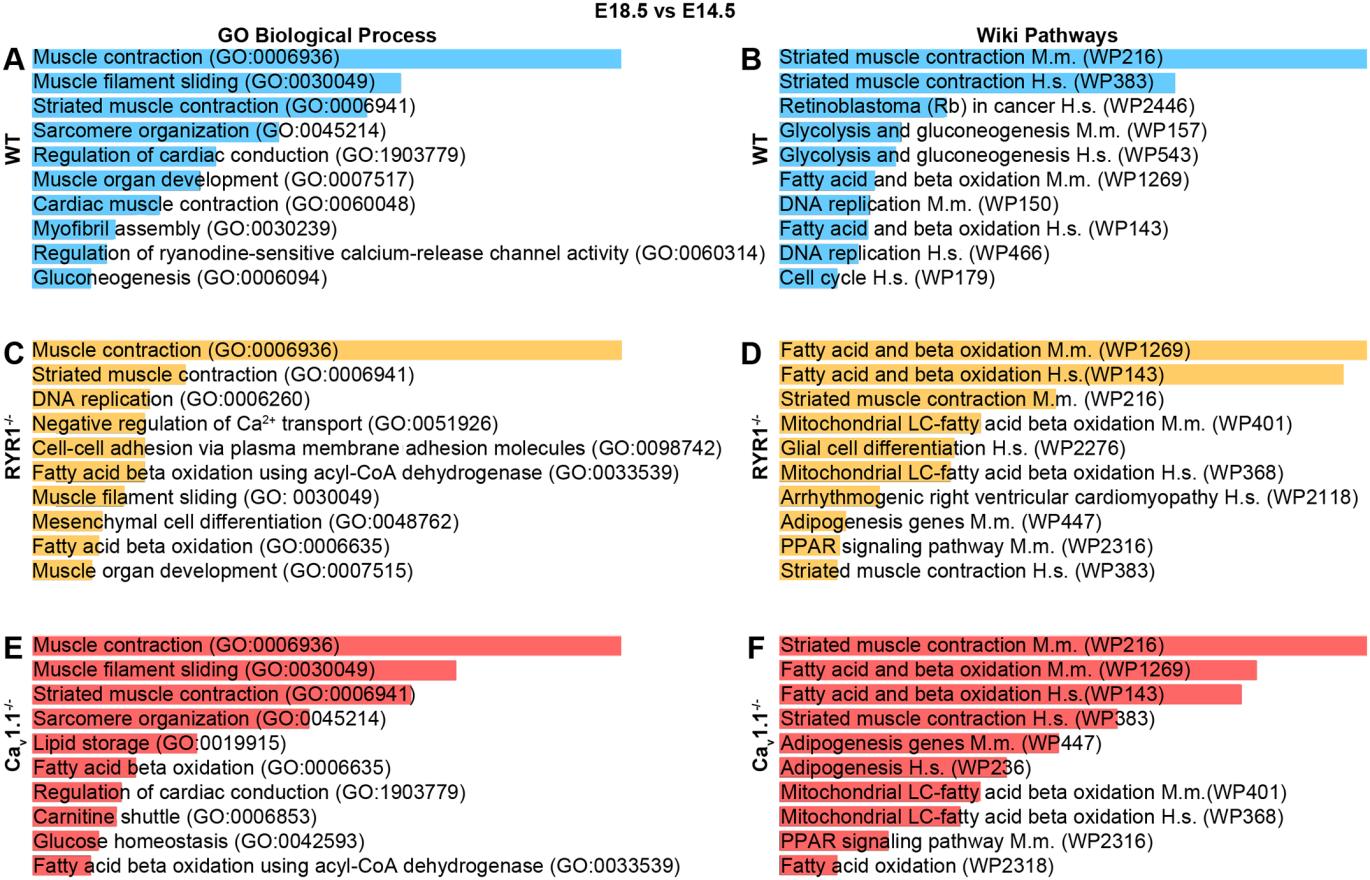
Analysis of all DEGs found in skeletal muscle development from E14.5 to E18.5. GO BP (A, C and E) and Wiki Pathways (B, D, F) enrichment analyses of all DEGs found in WT (A, B), RYR1^-/-^ (C, D) and Ca_v_1.1^-/-^ (E,F) from E14.5 (control) to E18.5. The ten most significantly enriched categories for each analysis are shown. Enrichment analyses (A–F) were performed via the Enrichr online tool [31], length of the bars is proportional to the significance (p-value).

In order to compare the skeletal muscle transcriptome of the E18.5 stage to that at E14.5, all DEGs identified in the E18.5 vs. E14.5, same-genotype comparison (WT vs. WT; RYR1^-/-^ vs. RYR1^-/-^; Ca_v_1.1^-/-^ vs. Ca_v_1.1^-/-^) were subjected to GO BP and WP enrichment analysis (Fig 11, S9 Table). In all three genotypes GO BP identified “Muscle contraction (GO:0006936)” as the most significantly involved biological process. In the case of E18.5 WT vs. E14.5 WT the GO BP enrichment analysis implicated additional processes related to muscle organization and contraction, which were only marginally represented in the analyses for RYR1^-/-^ or Ca_v_1.1^-/-^ muscle. On the other hand, both RYR1^-/-^ and Ca_v_1.1^-/-^ samples showed enrichment of DEGs involved in processes related to fatty acid metabolism and β-oxidation. Furthermore, RYR1^-/-^ samples also exhibited an enrichment of DEGs involved in “DNA replication (GO:0006260)”, “Negative regulation of calcium ion transport (GO:0051926)”, “Cell-cell adhesion via plasma-membrane adhesion molecules (GO:0098742)”, and “Mesenchymal cell differentiation (GO:0048762)”. Ca_v_1.1^-/-^ samples, on the other hand, showed a specific enrichment in “Lipid storage (GO:0019915)”, “Carnitine shuttle (GO:0006853)” and “Glucose homeostasis (GO:0042593)”. The WP enrichment analyses revealed “Striated muscle contraction Mus musculus (WP216)” as the most-significantly engaged process in WT and Ca_v_1.1^-/-^ samples, whereas in the RYR1^-/-^ samples “Fatty acid and beta oxidation Mus musculus (WP1269)/Homo sapiens (WP143)” were more significantly implicated. Additional pathways which were significantly enriched in WT samples were related to glucose metabolism, DNA replication and cell cycle, whereas the RYR1^-/-^ or Ca_v_1.1^-/-^ samples exhibited a more prominent enrichment in processes related to fat and energy metabolism.

### The E18.5 vs. E14.5 comparison reveals overlap but also genotype-specific DEGs between WT, RYR1^-/-^ and Ca_v_1.1^-/-^ limb skeletal muscle

**Fig 12:**
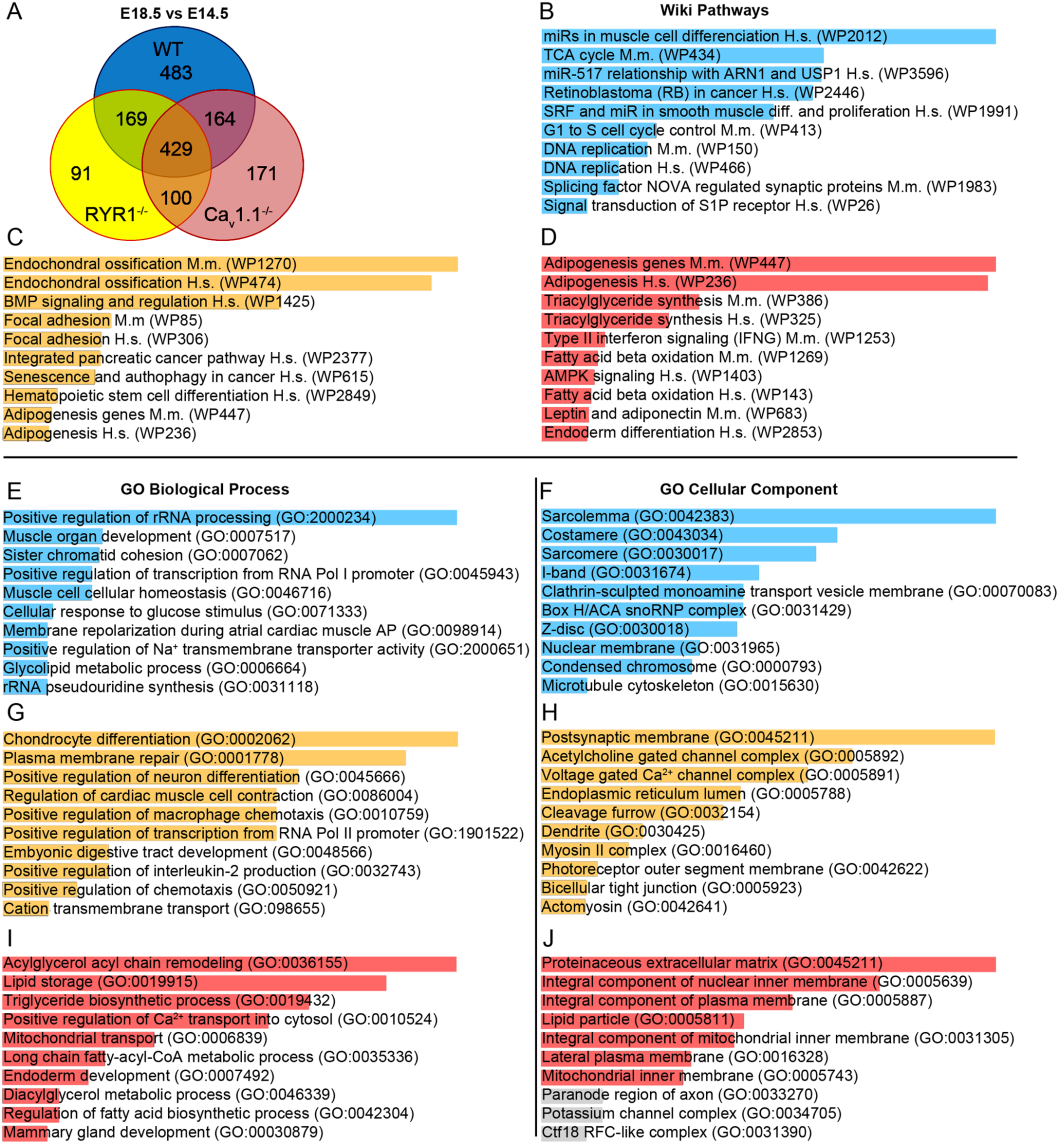
DEGs specific for the E14.5 to E18.5 development of WT, RYR1^-/-^ or Ca_v_1.1^-/-^ skeletal muscle. (A) A Venn diagram, showing the number of DEGs identified in the MA analyses at E18.5 compared to E14.5 in WT, RYR1^-/-^ and Ca_v_1.1^-/-^ limb skeletal muscle. Numbers in the overlapping and non-overlapping areas represent the amount of shared and not shared DEGs between genotypes, respectively. Wiki Pathways (B, C, D), GO BP (E, G, I) and GO CC (F, H, J) enrichment analyses of the DEGs found exclusively in WT (483 DEGs, blue charts), RYR1^-/-^ (91 DEGs, yellow charts) and Ca_v_1.1^-/-^ (171 DEGs, red charts) from E14.5 (control) to E18.5, respectively. The ten most significantly enriched categories for each analysis are shown. Enrichment analyses (B–J) were performed via the Enrichr online tool [31], length of the bars is proportional to the significance (p-value). Gray bars in (J) indicate a p-value ≥ 0.05.

One of our major objectives was to compare the global expression changes occurring from E14.5 to E18.5 in WT, RYR1^-/-^ and Ca_v_1.1^-/-^ skeletal muscle. Therefore, we inspected how many DEGs were shared and how many were specifically regulated in the development of WT, RYR1^-/-^ and Ca_v_1.1^-/-^ limb skeletal muscle (Fig 12A). This analysis revealed 429 common DEGs with changed expression levels from E14.5 to E18.5 in all examined genotypes, 169 DEGs shared between WT and RYR1^-/-^ samples, 164 DEGs shared between WT and Ca_v_1.1^-/-^ samples and 100 DEGs shared between RYR1^-/-^ and Ca_v_1.1^-/-^ samples. Moreover, 483 DEGs were specifically found only in the WT development, 91 DEGs—only in the RYR1^-/-^ and 171 DEGs—only in the Ca_v_1.1^-/-^ development.

To better understand which pathways, processes and structures were influenced by the DEGs of a particular genotype they were subjected to WP, GO BG and GO CC enrichment analyses (Fig 12B–12J, S10 Table). For the WT-specific DEGs the WP enrichment analysis highlighted “miRs in muscle cell differentiation Homo sapiens (WP2012)” as well as other miRNA-related pathways and pathways related to cell cycle and signal transduction. Analyzing the RYR1^-/-^-specific DEGs, the WP enrichment analysis identified endochondral ossification, BMP signaling and focal adhesion as significantly affected pathways, whereas in Ca_v_1.1^-/-^ these were pathways related to adipogenesis and lipid metabolism.

Similar results were obtained by the GO BP analysis, identifying “Positive regulation of rRNA processing (GO:2000234)” and several muscle- and cell cycle-related processes as highly enriched with WT-specific DEGs. “Chondrocyte differentiation (GO:0002062)”, among other developmental processes, was enriched with RYR1^-/-^-specific DEGs while “Acylglycerol acyl chain remodeling (GO:0036155)” and other lipid and fatty acid metabolic processes were enriched with Ca_v_1.1^-/-^-specific DEGs. The GO CC enrichment analysis indicated specific changes in the “Sarcolemma (GO:0042383)” and other muscle-specific structures like “Costamere (GO:0043034)”, “Sarcomere (GO:0030017)”, “I band (GO:0031674)”, and “Z disc (GO:0030017)” in the WT-specific DEGs analysis. In the GO CC enrichment analysis of the RYR1^-/-^-specific DEGs the structures “Postsynaptic membrane (GO:0045211)”, “Acetylcholine gated channel complex (GO:0005892)” and “Voltage-gated Ca^2+^ channel complex (GO:0005891)” had the highest significance; and the “Proteinaceous extracellular matrix (GO:0045211)”, “Integral component of nuclear inner membrane (GO:0005639)” and “Integral component of plasma membrane (GO:0005887)” were the top three cellular structures, enriched with Ca_v_1.1^-/-^-specific DEGs.

These results show, not unexpected [28], that the mutants recapitulate only a part of the transcriptomic changes associated with the development from E14.5 to E18.5 in WT skeletal muscle. However, the distinct transcriptomes of RYR1^-/-^ and Ca_v_1.1^-/-^ muscles and the resulting differences in the allocation of DEGs to cellular processes also imply, from a causative point of view, that there is more to it than the mere absence of contraction and of the associated mechanical movement. Our data thus suggest functions additional to excitation-contraction coupling, of the two Ca^2+^ channels during skeletal muscle development. Recent experiments reported by other groups suggest distinct, extra-contractile functions of RYR1 and Ca_v_1.1 in skeletal muscle development (detailed in Discussion).

### Differential expression of microRNAs (miRNA) during limb secondary myogenesis

**Fig 13:**
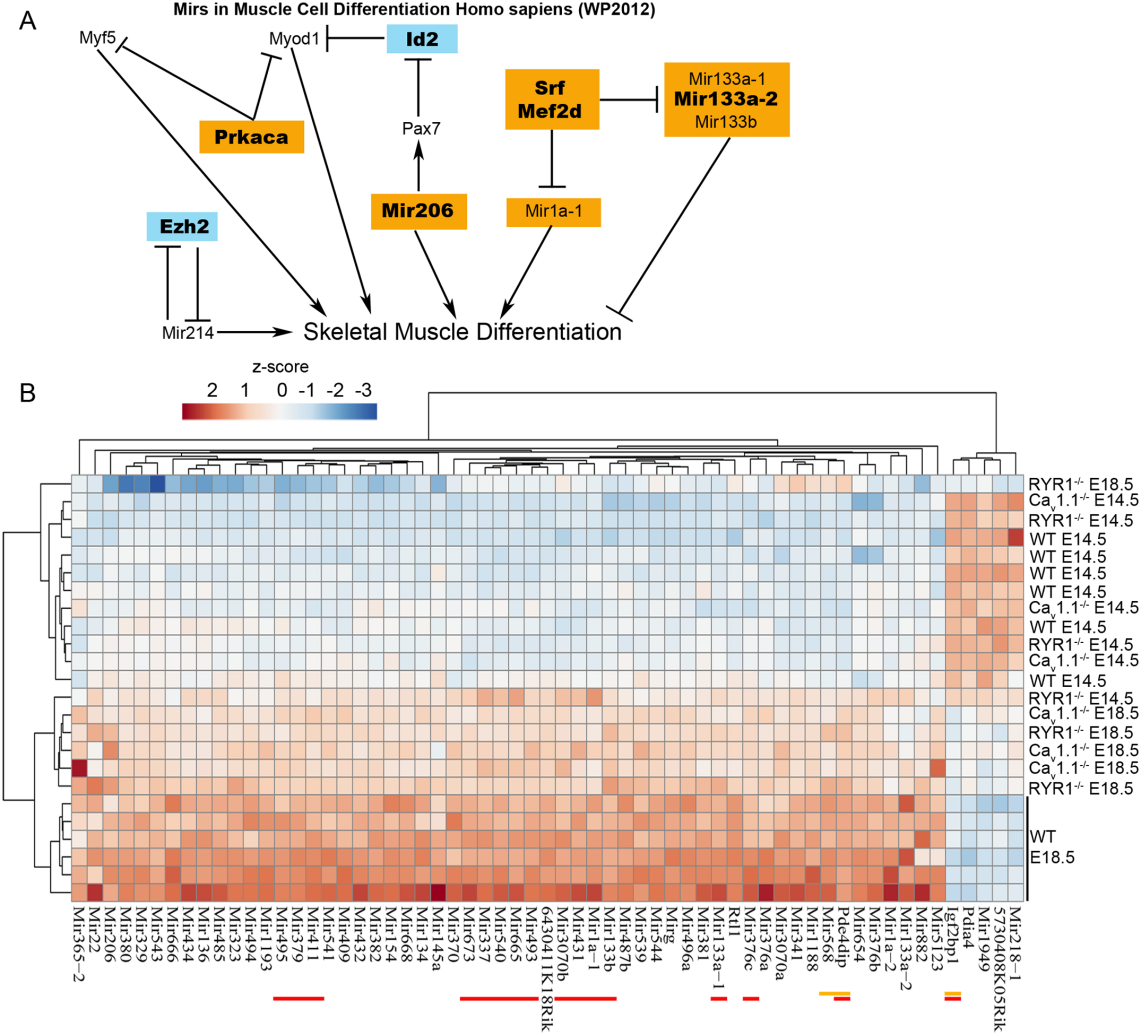
miRNAs identified by the MAs during WT skeletal muscle development. (A) Up (orange) and down (blue) regulated DEGs identified by the MAs for E18.5 vs. E14.5 (E14.5 is control) taking part in the Wiki pathway “Mirs in Muscle Cell Differentiation Homo sapiens (2012)”. DEGs regulated only in WT samples from E14.5 to E18.5 are shown in bold. (B) A heat map of all miRNAs, found to be differentially regulated at E18.5 compared to E14.5 in WT samples. Each row represents one biological replicate. miRNAs, found to be differentially regulated from E14.5 to E18.5 also in RYR1^-/-^ samples, are underlined in yellow, in the Ca_v_1.1^-/-^ samples in red, and in both RYR1^-/-^ and Ca_v_1.1^-/-^ samples in yellow and red, respectively. The heatmap was generated from the MAs intensity levels of each of the Mir genes via ClustVis [32].

Two of the pathways significantly enriched with DEGs in the WP analysis (Fig 12B), displayed changes in miRNA expression in E18.5 vs. E14.5 for WT but not for RYR1^-/-^ or Ca_v_1.1^-/-^ limb skeletal muscle, suggesting that miRNAs are part of the regulatory repertoire on which the two Ca^2+^ channels impart during secondary myogenesis. A further analysis of the DEGs participating in the “miRs in muscle cell differentiation Homo sapiens (WP2012)” pathway revealed 10 genes to be differentially expressed in WT samples and only 3 in RYR1^-/-^ or Ca_v_1.1^-/-^ samples from E14.5 to E 18.5 (Fig 13A). Among the DEGs found only in WT are genes encoding modulators of some of the canonical MRFs like *Myod*, *Myf5* and *Pax7*; as well as 2 muscle-specific miRNAs (Myomirs), Mir206 and Mir133a-2, both known to be involved in muscle differentiation [37,38]. These results prompted us to analyze the MA expression levels of all differentially regulated miRNAs detected in E18.5 vs. E14.5 comparisons in WT, RYR1^-/-^ and Ca_v_1.1^-/-^ samples (Fig 13B, Table 3). 61 miRNAs were differentially expressed in WT skeletal muscle, of which 16 were present also in the in Ca_v_1.1^-/-^ and 3 in the RYR1^-/-^ samples. Additionally, we found one differentially expressed miRNA in RYR1^-/-^ and 4 in Ca_v_1.1^-/-^, but not in WT muscle. A hierarchical clustering analysis displayed a clear grouping of the WT samples at E18.5 according to their miRNAs expression profiles (Fig 13B). A partial clustering was observed for the RYR1^-/-^ and Ca_v_1.1^-/-^ samples at E18.5 on one side, and all samples at E14.5 on the other, wherein one RYR1^-/-^ E18.5 sample was clustered closer to the E14.5 samples than to the other E18.5 samples. Notably, 56 miRNAs were upregulated and only 5 miRNAs were downregulated in the WT samples at E18.5 compared to E14.5. A similar tendency, but to a smaller extent, was observed for most RYR1^-/-^ and Ca_v_1.1^-/-^ samples, however for most miRNAs no significant changes in expression were detected. Apart from the Myomirs, the MAs identified at least another 22 miRNAs, implicated in muscle development and in various myopathies, to be differentially expressed in WT samples during secondary myogenesis (Table 3). Interestingly, 32 (i.e., 52% of all) of the identified miRNAs which were upregulated in E18.5 relative to E14.5, in WT have been found by others to be downregulated in ageing skeletal muscle [39], suggesting that these miRNAs might have important roles during skeletal muscle development and during subsequent adaptation or alteration. Moreover, 26 (43% of all) of the identified miRNAs originate from a miRNA cluster, located within the imprinted *Dlk-Dio3* genomic region on chromosome 12. This region might be of eminent importance for skeletal muscle development.

**Table 3:** miRNAs differentially regulated from E14.5 to E18.5 in WT, RYR1^-/-^ and Ca_v_1.1^-/-^.

Description	Gene Symbol	FC E18.5 vs. E14.5			Muscle-related functions	Reference
		WT	RYR1^-/-^	Ca_v_1.1^-/-^		
**Downregulated miRNAs**						
microRNA 1949	Mir1949	-3.25	-	-		
insulin-like growth factor 2 mRNA binding protein 1; microRNA 3063	Igf2bp1	-3.23	-3	-2.43		
protein disulfide isomerase associated 4; microRNA 704	Pdia4	-2.59	-	-		
microRNA 218–1	Mir218-1	-2.58	-	-	involved in muscle-bone communication and Wnt signaling	[40]
RIKEN cDNA 5730408K05 gene; microRNA 5136	5730408K05Rik	-2.1	-	-		
**Upregulated miRNAs**						
***MyoMirs***						
microRNA 206	Mir206	2.08	-	-	promotes myoblast entry into terminal differentiation	[37]
microRNA 133b	Mir133b	2.73	-	2.12	enhances myoblast proliferation	[38]
microRNA 133a-2	Mir133a-2	3.69	-	-	enhances myoblast proliferation	[38]
microRNA 1a-2	Mir1a-2	4.01	-	-	positive roles in muscle development	[37]
microRNA 133a-1	Mir133a-1	5.36	-	2.83	enhances myoblast proliferation	[38]
microRNA 1a-1	Mir1a-1	6.29	-	2.32	positive roles in muscle development	[37]
***miRNAs encoded in the Dlk-Dio3 genomic region***						
**microRNA 323**	**Mir323**	**2.22**	**-**	**-**		
**microRNA 668**	**Mir668**	**2.32**	**-**	**-**		
**microRNA 134; miRNA containing gene**	**Mir134**	**2.35**	**-**	**-**	**possibly targets *Pax7* and *Myf5***	[41]
**microRNA 485; miRNA containing gene**	**Mir485**	**2.4**	**-**	**-**		
**microRNA 494**	**Mir494**	**2.42**	**-**	**-**		
**microRNA 673**	**Mir673**	**2.48**	**-**	**2.24**		
**microRNA 544**	**Mir544**	**2.5**	**-**	**-**		
**microRNA 382**	**Mir382**	**2.53**	**-**	**-**	**increased in Becker muscular dystrophy**	[42]
**microRNA 666**	**Mir666**	**2.55**	**-**	**-**		
**microRNA 539**	**Mir539**	**2.58**	**-**	**-**	**disregulated in DMD dogs**	[43]
**microRNA 541**	**Mir541**	**2.71**	**-**	**-**		
**microRNA 381**	**Mir381**	**2.9**	**-**	**-**	**implicated in muscle differentiation**	[44]
**microRNA 487b**	**Mir487b**	**2.98**	**-**	**-**	**delays myogenic differentiation in C2C12**	[45]
**microRNA 431**	**Mir431**	**2.98**	**-**	**2**	**promotes differentiation and regeneration of old skeletal muscle**	[46]
**miRNA containing gene; microRNA 410; microRNA 412; microRNA 369**	**Mirg**	**3**	**-**	**-**		
**microRNA 409**	**Mir409**	**3.17**	**-**	**-**	**upregulated in nemaline myopathy**	[47]
**microRNA 495**	**Mir495**	**3.5**		**2.4**		
**microRNA 496a**	**Mir496a**	**3.51**	**-**	**-**		
**microRNA 379**	**Mir379**	**3.8**	**-**	**2.28**		
**microRNA 411**	**Mir411**	**3.8**	**-**	**2.64**	**involved in myogenic proliferation in FSHD and rhabdomyosarcoma**	[48,49]
microRNA 341	Mir341	2.15	-	-		
microRNA 380	Mir380	2.57	-	-		
microRNA 654	Mir654	2.85	-	-		
microRNA 154	Mir154	3.2	-	-	downregulated by TNF-α in skeletal muscle differentiation	[50]
microRNA 376c	Mir376c	3.35	-	3.34		
microRNA 376b	Mir376b	3.69	-	-	a role in cardioprotection	[51]
***Other miRNAs implicated in skeletal muscle development***						
**RIKEN cDNA 6430411K18 gen; microRNA 127; microRNA 433**	**6430411K18Rik**	**2.78**	**-**	**-**	**enhances myogenic cell differentiation**	[52]
**microRNA 376a**	**Mir376a**	**2.81**	**-**	**-**	**involved in skeletal muscle development**	[53]
**microRNA 665**	**Mir665**	**2.92**		**2.04**	**associated in secondary myogenesis in pigs**	[54]
**microRNA 136**	**Mir136**	**3.41**	**-**	**-**	**downregulated in mouse skeletal muscle after birth**	[41]
**microRNA 434**	**Mir434**	**3.55**	**-**	**-**	**influences AChRs expression in rat hind limb**	[55]
**microRNA 540**	**Mir540**	**3.56**	**-**	**2.05**	**induces hypertrophy in C2C12**	[56]
microRNA 432	Mir432	2.04	-	-	regulates myoblast proliferation and differentiation	[57]
microRNA 365–2	Mir365-2	2.04	-	-	putative inhibition of myogenic differentiation in C2C12	[58]
microRNA 145a	Mir145a	2.1	-	-	promotes myoblast differentiation	[59]
microRNA 22; Mir22 host gene (non-protein coding); TLC domain containing 2	Mir22	2.24	-	-	up-regulated during myocyte differentiation	[60]
microRNA 5123	Mir5123	2.29	-	-	upregulated in ageing muscle	[61]
***miRNAs not yet described in skeletal muscle development***						
**microRNA 1193**	**Mir1193**	**2.01**	**-**	**-**		
**microRNA 329**	**Mir329**	**2.15**	**-**	**-**		
**microRNA 1188**	**Mir1188**	**2.16**	**-**	**-**		
**microRNA 543**	**Mir543**	**2.43**	**-**	**-**		
**retrotransposon-like 1; microRNA 3071**	**Rtl1**	**3.54**	**-**	**-**		
**microRNA 337**	**Mir337**	**5.61**	**-**	**3.06**		
microRNA 882	Mir882	2.01	-	-		
microRNA 3070b	Mir3070b	2.81	-	2.01		
microRNA 3070a	Mir3070a	2.83	-	-		
microRNA 370	Mir370	3.02	-	-		
microRNA 493	Mir493	3.76	-	2.52		
microRNA 568; zinc finger and BTB domain containing 20	Mir568	4.41	2.65	-		
phosphodiesterase 4D interacting protein (myomegalin); microRNA 7225	Pde4dip	4.49	4.1	3.79		
**miRNAs found exclusively in RYR1**^**-/-**^ **skeletal muscle development (E18.5 vs. E14.5)**						
microRNA 125b-1	Mir125b-1	-	-2.11	-		
**miRNAs found exclusively in Ca**_**v**_**1.1**^**-/-**^ **skeletal muscle development (E18.5 vs. E14.5)**						
microRNA 205; RIKEN cDNA 4631405K08 gene	Mir205	-	-	-2.38		
microRNA 669d	Mir669d	-	-	-2.07		
glutamate-ammonia ligase (glutamine synthetase); microRNA 8114	Glul	-	-	2.14		
microRNA 130a	Mir130a	-	-	2.28		

miRNAs reported as downregulated in ageing skeletal muscle are written in bold text [39]. The FCs of miRNAs not detected as differentially regulated in some of the conditions are marked as “-”.

## Discussion

In this study we analyzed the histological and transcriptomic changes occurring in the developing limb skeletal muscle in the absence of RYR1 or Ca_v_1.1. We have previously shown that, besides the known fact that homozygous loss of RYR1 (RYR1^-/-^) is associated with severely altered skeletal muscle structure at E18.5, gene expression at this later stage displays a characteristic signature [28]. The spectrum of severity resulting from mutations in RYR1 is demonstrated, for instance, in skeletal muscles from patients suffering from diseases like atypical periodic paralysis and myalgia [62], and by the lethal multiple pterygium syndrome [63].

Here we demonstrate that skeletal muscle from both homozygous RYR1^-/-^ and Ca_v_1.1^-/-^ mice exhibits moderate but discrete morphological and transcriptomic changes already at E14.5, suggesting that RYR1 and Ca_v_1.1 are involved in the embryonic development and primary myogenesis. Histological abnormalities and gene expression changes of these animals become much more overt at E18.5, demonstrating the vital importance of the two Ca^2+^ channels during fetal development of muscle. The latter term does not only refer to muscle cells or fibers in the narrower sense, but also comprises the other constituents of the muscle organ like nerves, blood vessels and connective tissue. As these structures take part in the normal development and the function of skeletal muscle, it is not only unavoidable but also desirable to have their transcriptomic impact integrated in the present analysis.

A first surprise in our analysis was to find that among the “early” DEGs, i.e. at E14.5, only 2 were shared by the homozygous mutants. Apart from these, the DEGs of RYR1^-/-^ and Ca_v_1.1^-/-^ samples at this early stage were associated with different biological processes (Fig 9), suggesting that RYR1 and Ca_v_1.1 have distinct roles during early myogenesis. E14.5 is approximately the stage at which the mouse embryos first begin to move [11]. Thus, although the cooperative action of both Ca^2+^ channels is required for proper ECC, they probably also exert additional, non-contractile functions during muscle development. At E14.5 the absence of RYR1 seems to negatively influence neuron differentiation and / or muscle innervation, whereas the lack of Ca_v_1.1 is already linked to downregulation of genes involved in the muscle’s contractile machinery. In cell culture, Ca_v_1.1 is expressed prior RYR1 [64] where it has been shown to lead to activation of phospholipase C (PLC), leading to Ca^2+^ influx from the SR through the inositol 1,4,5-triphosphate receptor (IP3R) [65]. The IP3R-mediated Ca^2+^ transients have been implicated in myogenesis and in additional signaling paths [66,67], hence some of the Ca_v_1.1^-/-^ specific transcriptomic changes are probably caused by an impaired activation of IP3Rs. Alternatively (or additionally), the distinct transcriptomic changes in the mutants at E14.5 may also be connected to Ca^2+^ influx into the cells from the extracellular space through the embryonic (Δ29) Ca_v_1.1 splice variant, which has been linked to acetylcholine receptor (AChR) pre-patterning of developing skeletal muscle [34]. We observed a higher ratio of Δ29 to full-length Ca_v_1.1 mRNA in RYR1^-/-^ compared to WT skeletal muscle at E14.5, which might be accompanied by an increased, Ca_v_1.1-mediated Ca^2+^ influx into these muscles upon spontaneous or motor neuron-caused depolarization. At E18.5 the ratio of the Δ29 to full-length Ca_v_1.1 mRNA was unchanged in RYR1^-/-^ compared to WT samples, but the total Ca_v_1.1 mRNA levels were decreased by 2-fold in the RYR1^-/-^ samples, which is in line with previous reports [29,35,36]. In contrast, the complete absence of the dihydropyridine receptor in Ca_v_1.1^-/-^ muscle, with the consequence of absent voltage–dependent activation of RYR1 and IP3R, may explain the higher number of DEGs at both E14.5 and E18.5 in these mutants, as well as their more severe muscle phenotype. Interestingly, it has recently been demonstrated in a mouse model expressing exclusively a non-conducting Ca_v_1.1, that the absence of Ca_v_1.1-mediated Ca^2+^ influx does neither affect skeletal muscle development, contractile properties and contractile protein expression, nor the normal phenotype, fertility and longevity of these animals [68]. Taking these recent observations into account, the majority of morphological and transcriptomic alterations we find in our study in Ca_v_1.1^-/-^ limb skeletal muscle most likely are not the consequence of absent Ca^2+^ influx through Ca_v_1.1, but would rather be caused by the lack of activation of RYR1- and/or IP3R-mediated Ca^2+^ release. Additionally, the physical absence of Ca_v_1.1 as critical element for interactions within the macromolecular EC coupling apparatus could contribute to these deteriorations.

The serious alterations in skeletal muscle gross structure and histology of the mutants at E18.5 (as compared to E14.5; Fig 2) are in line with the 8 to 10-fold greater number of DEGs in RYR1^-/-^ and Ca_v_1.1^-/-^ vs. WT at this later stage (Table 2). But not only the number of DEGs in both RYR1^-/-^ and Ca_v_1.1^-/-^ is considerably greater at E18.5, there is also, in contrast to E14.5, a significant overlap in the identity of DEGs in both mutants. This indicates that the absence of mechanical movement and the lack of associated Ca^2+^ signaling lead to transcriptomic changes ultimately shared by both homozygous mice models, and probably also by developmental paralysis models in general (as discussed in [28]). Accordingly, at E18.5 multiple genes encoding proteins associated with the contractile machinery were downregulated in the mutants (Fig 10), possibly through a negative feedback loop due to the lack of mechanical loading. Some of these DEGs encode thick (*Myl2*, *Myh13*, *Myl9*, *Myh7*, *Myl6b*) and thin (*Tpm3*, *Myom1*, *Myom2*) filament proteins, Z-disc proteins (*Csrp3*, *Rcsd1*, *Actn2*, *Tcap*), as well as proteins taking part in the structure of costameres (*Myof*, *Ankrd2*, *Dmd*, *Sgca*, *Myot*) and ion channels (*Cacna1h*, *Kcne1l*, *Kcnma1*, *Ryr1*, *P2rx6*, *Cacna1s*, *Cacng1*, *Kcnq1*, *Clcn1*). These structures, especially the Z-disc and the costamere, play important roles in signal transmission between the ECM, the sarcolemma and the myofibrils, in the process of mechanotransduction [69,70]. Mechanical stimuli have been long known to contribute to muscle development and hypertrophy by activating an intricate network of downstream signaling cascades which include integrins, G-protein coupled receptors, the nuclear factor of activated T cells (NFAT), PI3K-Akt and MAPK pathways [15,71,72]. These signaling pathways are also regulated by changes in the cytosolic Ca^2+^ concentration which in turn is affected by various mechanosensitive pathways [16]. Thus, it is not surprising that the absence of RYR1 or Ca_v_1.1 –each indispensable for the rapid Ca^2+^ release that triggers muscle contraction—causes changes in the expression of multiple genes, involved in these major signaling pathways (S4 Table).

Our study identified a higher number of DEGs for WT in the E18.5 vs. E14.5 comparison than for either mutant (Table 2). Thus, a considerable fraction of the DEGs found in the E18.5 mutants vs. WT comparison, might emerge from a failure in the two mutants to activate the normal developmental expression program. This is reflected by the processes associated with the DEGs emerging from the E18.5 vs. E14.5 comparison (Fig 12). Specifically, multiple genes involved in the cell cycle control (*Prim2*, *Ccnd2*, *Mcm7*, *Cdk4*, *Mcm2*, *Plk1*, *Mad2l1*) were downregulated only in WT samples—a typical sign for terminal differentiation. Moreover, many genes encoding proteins of the muscle contractile machinery were found to be upregulated from E14.5 to E18.5 exclusively in WTs (Fig 12F, S10 Table); or to be upregulated with a much smaller FC in the mutants like *Mybpc2*, *Ckmt2*, *Myh2*, *Myh4* and *Mylk2* (S1 Table). These observations strongly imply that the secondary myogenesis, normally involving an increased level and organization of contractile structures, and accompanied by the exit from the cell cycle during fetal development, is impaired in RYR1^-/-^ and Ca_v_1.1^-/-^ limb skeletal muscle.

We have further detected a differential expression of at least 61 miRNAs in WT limb skeletal muscle from E14.5 to E18.5, with only a few of them undergoing a parallel regulation in both mutants (Table 3). Many miRNAs have been found to be potent regulators of gene expression in general and of muscle differentiation, in particular [73]. Therefore, the altered miRNA developmental patterns in RYR1^-/-^ and Ca_v_1.1^-/-^ limb skeletal muscle are likely to have contributed to the observed transcriptomic changes. The vast majority of differentially expressed miRNAs identified in the WT showed an upregulation at E18.5 vs. E14.5. Interestingly, more than half of them were reported to be also downregulated in ageing skeletal muscle (Table 3) [39], suggesting important roles for these miRNAs in myogenesis and in skeletal muscle maintenance. In this respect, the *Dlk-Dio3* genomic region—a miRNA megacluster encoding more than 50 miRNAs—appears to be of eminent importance [74]: 26 of the developmentally upregulated miRNAs we found in WT skeletal muscle originate from this region. Also, reduced expression of miRNAs from the *Dlk-Dio3* cluster has been implicated in the ageing process in gastrocnemius muscles [75], and myostatin deficiency has been shown to lead to a transcriptional activation of this locus [56]. Only 6 of the miRNAs upregulated in WT (E18.5 vs. E14.5) were also upregulated in Ca_v_1.1^-/-^ muscle and none in RYR1^-/-^ muscle (Table 3). Thus, our findings indicate that the increased expression level of multiple miRNAs from the *Dlk-Dio3* genomic region is a significant contributor to secondary myogenesis and that muscle contraction probably drives their expression.

In the E18.5 vs. E14.5 comparison we observed sets of DEGs exclusively regulated in either RYR1^-/-^ or Ca_v_1.1^-/-^ limb skeletal muscle (Fig 12). The cellular processes and structures affected by these DEGs in the RYR1^-/-^ samples were related to bone, cartilage and neuron differentiation, focal adhesion and ion channels; whereas in the Ca_v_1.1^-/-^ samples these were predominantly processes linked to adipogenesis and lipid metabolism. Given the critical signaling role of [Ca^2+^]_i_, these differences may originate from the different resting [Ca^2+^]_i_ in both mutants—resting Ca^2+^ levels were found to be lower than in WT in cultured RYR1^-/-^ myotubes and higher than in WT in Ca_v_1.1^-/-^ myotubes [76–78]. These results have been accounted for by a model in which Ca_v_1.1 is necessary for inhibiting spontaneous Ca^2+^ leak trough RYR1. This model would also explain the upregulation of *Musk*, *Chrnd* and *Chrng*, detected only during development of RYR1^-/-^ skeletal muscle, as these genes are negatively regulated by increased [Ca^2+^]_i_ and in turn regulate proper neuromuscular synaptic pre-patterning [79]. Additionally, as discussed above, the absence of Ca_v_1.1 may also lead to explicit differences in gene expression because of lack or reduction of the IP3R-mediated Ca^2+^ transients. The evident changes in the expression of genes associated with lipid metabolism would also suggest alterations in mitochondrial function and / or mitochondrial Ca^2+^ uptake in Ca_v_1.1^-/-^ skeletal muscle. The latter could be a contributing factor to the increased levels of apoptosis observed in Ca_v_1.1^-/-^ skeletal muscle at E14.5, however, an increased lipotoxicity is also thinkable in this respect [80].

Unexpectedly, unlike the heterozygous RYR1^+/-^ skeletal muscle, which does not display obvious alterations with respect to WT, heterozygous Ca_v_1.1^+/-^ skeletal muscle is characterized by morphological aberrations at both E14.5 and E18.5. Such a phenotype has not been reported previously for Ca_v_1.1^+/-^ skeletal muscle, which has been regarded as equivalent to WT [81–84]. Nevertheless, an altered mandible development has been described in Ca_v_1.1^+/-^ animals [85], indicating that a precise gene dosage of Ca_v_1.1 may also be necessary for a stable muscle development. However, very few DEGs were detected between Ca_v_1.1^+/-^ and WT skeletal muscles at both E14.5 and E18.5 (Table 2).

Taken together, our findings provide important information about the changes occurring in the transcriptomic landscape of limb skeletal muscle during secondary myogenesis in mouse. We have shown that absence of RYR1 or Ca_v_1.1 leads to partially severe histological changes in limb skeletal muscle both at the beginning (E14.5) and, more so, the end (E18.5) of secondary myogenesis. At both time points the global gene expression profiles of RYR1- and Ca_v_1.1-deficient muscle exhibit significant changes, affecting an extensive array of genes related to structure and to key signaling pathways. At E14.5 we observed fewer but distinct DEGs in each mutant, whereas at E18.5 the expression changes in both mutants became vast and partially converged. The significantly higher number of affected genes at E18.5 together with the suppression of myogenic progression from E14.5 to E18.5 in both mutants, indicate that presence of RYR1 and Ca_v_1.1 is essential during secondary myogenesis. Thus, we hypothesize that RYR1 and Cav1.1, beyond their critical role in skeletal muscle ECC, have also important, partially discrete roles in both embryonic and fetal skeletal muscle development. Future work will elucidate the molecular mechanisms by which RYR1 and Ca_v_1.1 influence skeletal muscle development.

## Supporting information

S1 Fig Ca_v_1.1 splice variants in WT and RYR1^-/-^ skeletal muscle. Original photographs of agarose gels used for the analysis of PCR products of the full-length (343 bp) and Δ29 (286 bp) Ca_v_1.1 splice variants in WT (A), and in RYR1^-/-^ (B) animals at E14.5 (A and B, lanes 1–6) and E18.5 (A and B, lanes 8–13). (A and B) Lane 7—O’Gene Ruler Mix DNA ladder.
(TIF)

S2 Fig RNA agarose gels. The integrity of the RNA samples used in the MA analyses was evaluated by subjecting 250 ng or 500 ng of each sample to electrophoretic runs on 2% agarose gels next to 2 μl of RiboRuler High Range RNA Ladder (Thermo Fisher Scientific). The genotypes of the mice are represented as follows: +/+ stands for WT, +/-—for heterozygous mutant and -/-—for homozygous mutant of the RYR1 and Ca_v_1.1 lines, respectively. The numbers 1–3 represent the individual biological replicates (fetuses); “n.” stands for samples that were not used in the MAs.
(TIF)

S3 Fig qRT-PCR analyses of putative endogenous controls. The relative expression levels of *Gapdh*, *Actb*, *Rplp0*, *Uba52* and *CytB* (used as endogenous control) were measured via qRT-PCRs for WT E18.5 vs. E14.5 samples (A), as well as for RYR1^-/-^ vs. WT (B and C) and for Ca_v_1.1^-/-^ vs. WT (D and E) at E14.5 and E18.5. Expression levels of control samples (blue bars) were set to 1. Statistical *t*-tests were performed for each gene, ***represents a p-value ≤ 0.001. Error bars are S.E.M.
(TIF)

S1 Table All detected DEGs from the MAs from all performed comparisons. (XLSX)

S2 Table GO BP enrichment analyses of RYR1^-/-^ or Ca_v_1.1^-/-^ vs. WT at E14.5. (XLSX)

S3 Table DEGs for heatmaps of RYR1^-/-^ or Ca_v_1.1^-/-^ vs. WT at E14.5. (XLSX)

S4 Table DEGs in RYR1^-/-^ or Ca_v_1.1^-/-^ vs. WT at E18.5 involved in signaling pathways. (XLSX)

S5 Table GO BP enrichment analyses of RYR1^-/-^ or Ca_v_1.1^-/-^ vs. WT at E18.5. (XLSX)

S6 Table DEGs for “Muscle contraction” heatmap of RYR1^-/-^ and Ca_v_1.1^-/-^ vs. WT at E18.5. (XLSX)

S7 Table DEGs for “Extracellular Matrix Organization” heatmap of RYR1^-/-^ vs. WT at E18.5. (XLSX)

S8 Table DEGs for “Acylglycerol Metabolic Process” heatmap of Ca_v_1.1^-/-^ vs. WT at E18.5. (XLSX)

S9 Table GO BP and WP analyses of all DEGs in WT, RYR1^-/-^ or Ca_v_1.1^-/-^ for E18.5 vs. E14.5. (XLSX)

S10 Table GO BP and WP analyses of unique DEGs in WT, RYR1^-/-^ or Ca_v_1.1^-/-^ for E18.5 vs. E14.5. (XLSX)
