# Supplementary figures and images for: Distinct transcriptomic changes in E14.5 mouse skeletal muscle lacking RYR1 or Cav1.1 converge at E18.5
Source: PLoS One. 2018 Mar 15;13(3):e0194428. doi: 10.1371/journal.pone.0194428 (PMC5854361; doi:10.1371/journal.pone.0194428)

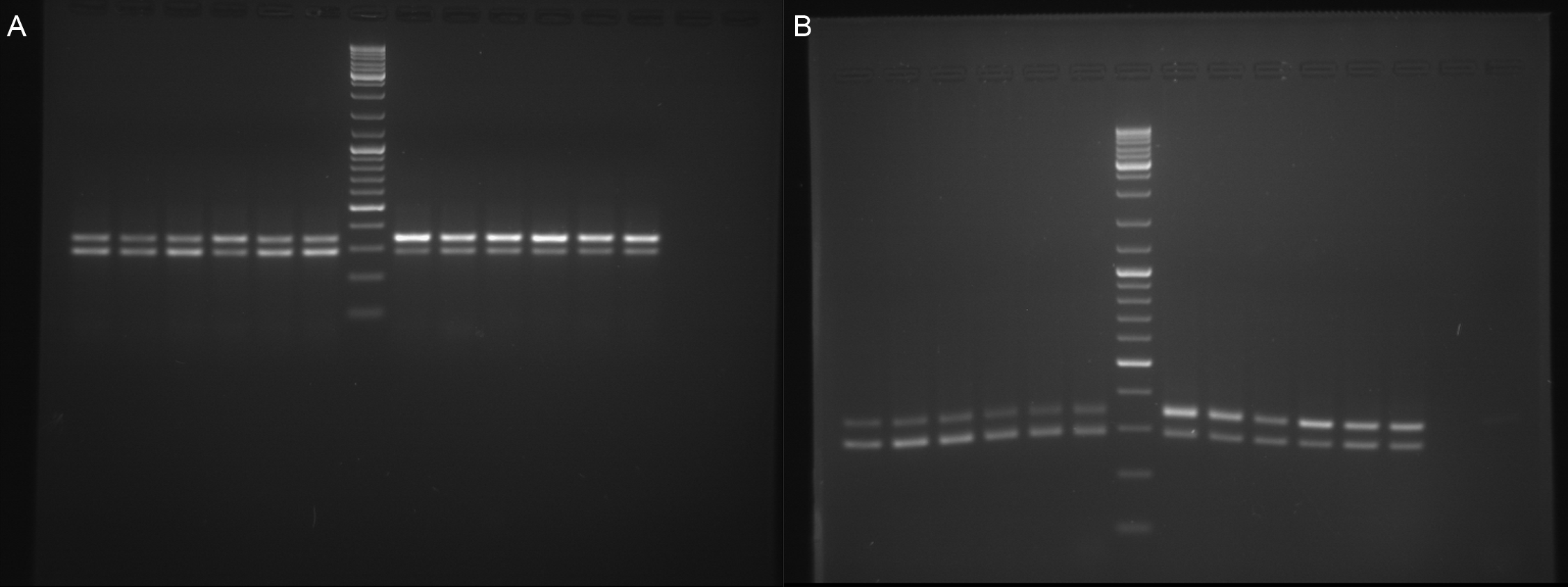

Supplement: S1 Fig — Original photographs of agarose gels used for the analysis of PCR products of the full-length (343 bp) and Δ29 (286 bp) Cav1.1 splice variants in WT (A), and in RYR1-/- (B) animals at E14.5 (A and B, lanes 1–6) and E18.5 (A and B, lanes 8–13). (A and B) Lane 7—O’Gene Ruler Mix DNA ladder. (TIF) [file pone.0194428.s001.tif]

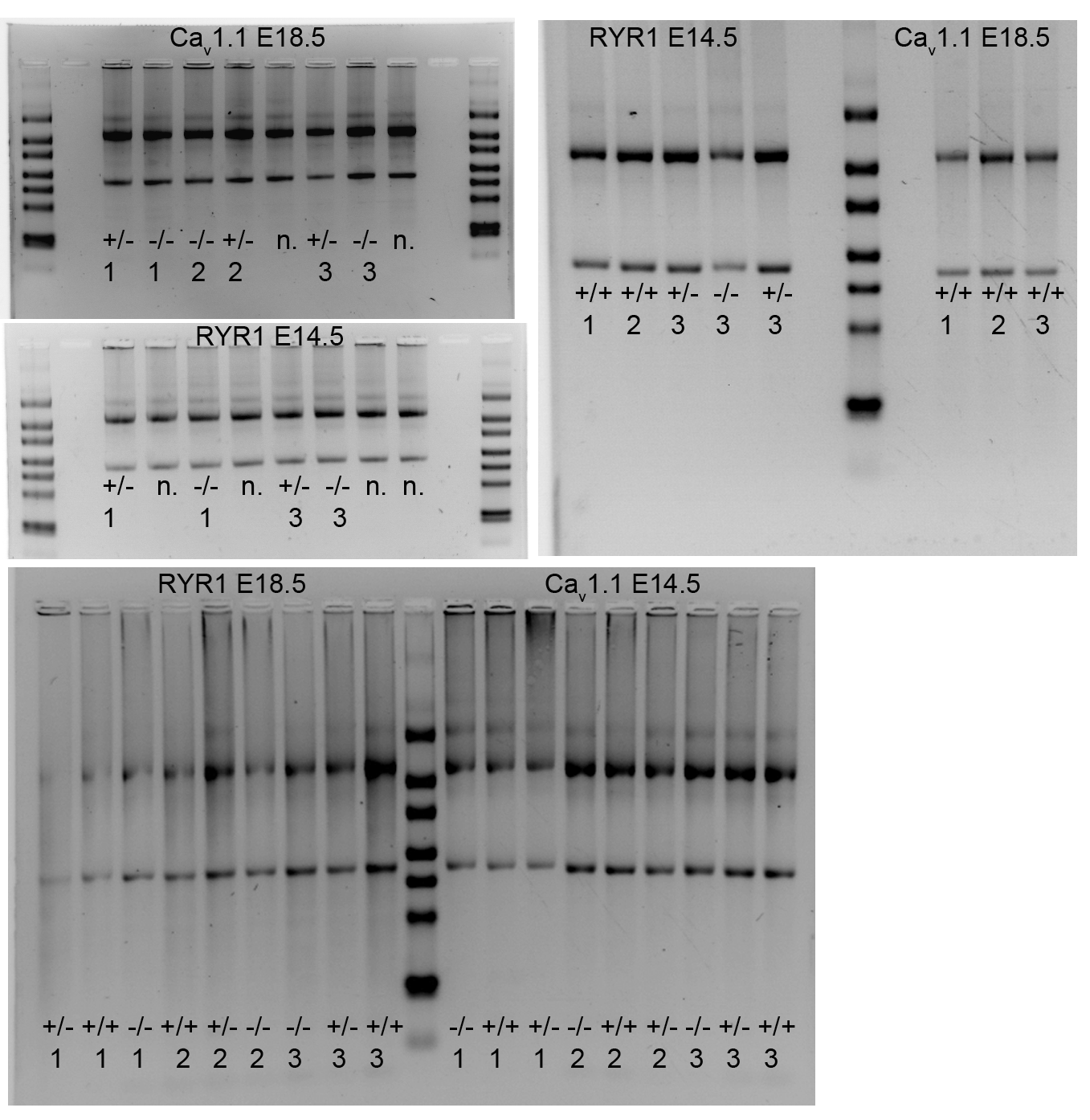

Supplement: S2 Fig — The integrity of the RNA samples used in the MA analyses was evaluated by subjecting 250 ng or 500 ng of each sample to electrophoretic runs on 2% agarose gels next to 2 μl of RiboRuler High Range RNA Ladder (Thermo Fisher Scientific). The genotypes of the mice are represented as follows: +/+ stands for WT, +/-—for heterozygous mutant and -/-—for homozygous mutant of the RYR1 and Cav1.1 lines, respectively. The numbers 1–3 represent the individual biological replicates (fetuses); “n.” stands for samples that were not used in the MAs. (TIF) [file pone.0194428.s002.tif]

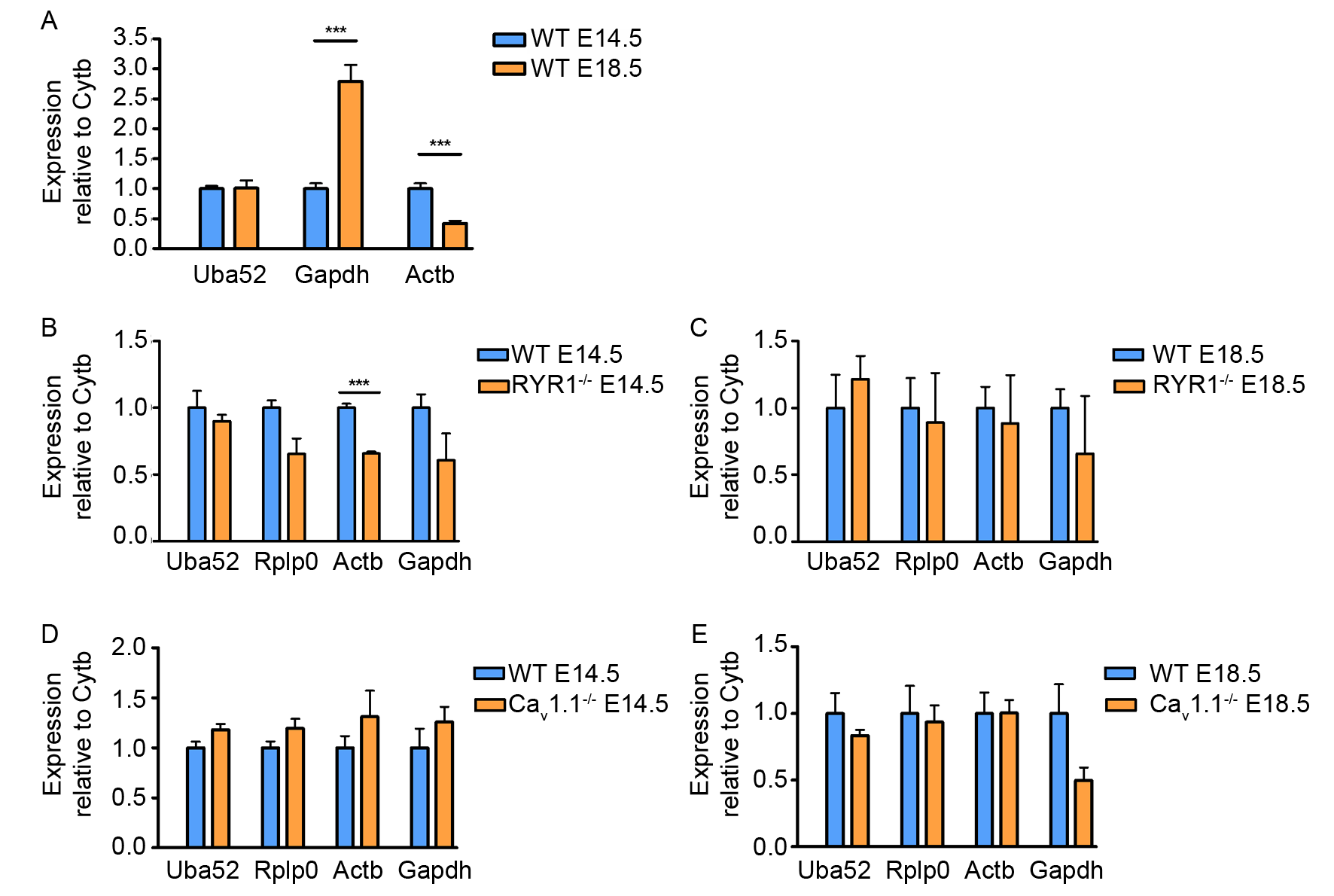

Supplement: S3 Fig — The relative expression levels of Gapdh, Actb, Rplp0, Uba52 and CytB (used as endogenous control) were measured via qRT-PCRs for WT E18.5 vs. E14.5 samples (A), as well as for RYR1-/- vs. WT (B and C) and for Cav1.1-/- vs. WT (D and E) at E14.5 and E18.5. Expression levels of control samples (blue bars) were set to 1. Statistical t-tests were performed for each gene, ***represents a p-value ≤ 0.001. Error bars are S.E.M. (TIF) [file pone.0194428.s003.tif]
